# Investigating the CPL‐Structure Dependence in Arylalkynylpyridyl—Triazacyclononane Sensitized Nine‐Coordinate Europium(III) Complexes

**DOI:** 10.1002/chir.70145

**Published:** 2026-09-07

**Authors:** Artemijs Krimovs, Robert Pal

**Affiliations:** ^1^ Department of Chemistry Durham University Durham DH1 3LE UK

## Abstract

A series of novel arylalkynylpyridyl‐triazacyclononane sensitized macrocyclic europium(III) complexes (EuL) was prepared, and their circularly polarized luminescence characterized in terms of the spectral profile and dissymmetry factor (*g*_lum_). The series was engineered based on previously reported isostructural complexes by a controlled introduction of structural modifications at different positions with respect to the coordination environment of the emitting europium(III) center. It was established that only those structural features of the macrocyclic ligand that were proximal to the europium(III) center affected CPL properties. These findings could help shape the development of a new class of purpose‐built EuL complexes compatible with CPL‐unaffecting complex‐stage functionalization to introduce advantageous structural features for applications in life and material sciences.

## Graphical abstract

A series of novel, systematically modified macrocyclic europium(III) complexes was prepared, and their circularly polarized luminescence (CPL) characterized in terms of the spectral profile and dissymmetry factor (*g*_lum_) to investigate CPL vs. structure relationship.

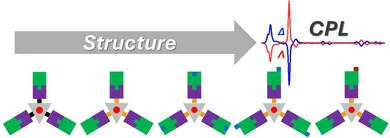

## 1 Introduction

**FIGURE 1:**
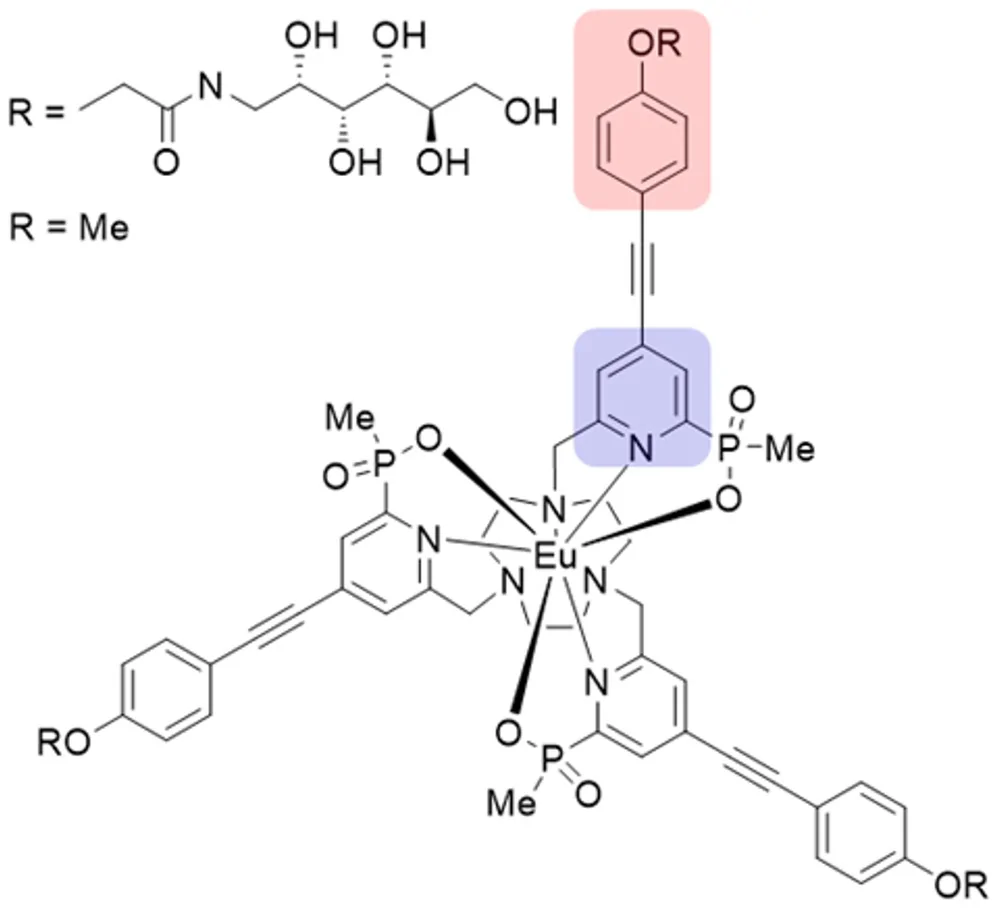
Examples of two EuL‐type complexes previously reported as luminescent dyes for bioimaging [1, 2, 3].

Arylalkynylpyridyl—triazacyclononane sensitized macrocyclic *quasi*‐*C*_3_ symmetric europium(III) complexes (EuL) have been long known for their outstanding brightness, low toxicity, and large pseudo‐Stokes shift, which are advantageous for bioimaging application. Their structure comprises a 1,4,7‐triazacyclononane (TACN)—based macrocyclic ligand (L) with three light absorbing chromophores that facilitate sensitization of the emissive europium(III) center (Figure 1). The chromophores contain electron‐rich aryl (Figure 1—red) and electron‐depleted pyridyl (Figure 1—blue) groups conjugated via an alkyne. From previous work, it was determined that such chromophore structure facilitates internal charge transfer (ICT) states that can be populated via photoexcitation (PE) [4]. This results from the highest occupied molecular orbital (HOMO) localizing on the electron‐rich aryl, whereas the lowest unoccupied molecular orbital (LUMO)—on the electron‐depleted pyridyl groups [5]. The two are spatially separated by a linear alkyne linker, resulting in a substantial displacement of charge upon PE producing high molar extinction coefficients (*ε*) of ~20,000 M^−1^ cm^−1^ per each chromophore (~60,000 M^−1^ cm^−1^ in total) [6]. Another benefit of sensitization via an ICT is the ability to bathochromically (red) shift the absorption maximum towards a longer wavelength ultraviolet (UV) range with electron‐donating substituents on the aryl group [6]. This shifts the absorption maximum towards commercially available 355 nm Nd:YAG laser (3rd harmonic) or 365 nm light emitting diode (LED) for applications in both bioimaging [1, 2, 3, 7] and security [8, 9, 10] authentication. In addition, efficient sensitization mechanism in EuL‐type materials results in relatively high values of photoluminescence quantum yield (*Φ*) of ~30%–40% [11]. Another benefit of EuL is their modular synthesis that facilitates their versatility, because the macrocyclic ligand precursors can be independently modified prior to complex formation.

## 2 Structure‐CPL Relationship in EuL Materials

EuL architecture provides a great platform for designing bright functional luminescent dyes, which produce strong circularly polarized luminescence (CPL) in their enantiopure form. Apart from excellent photophysical properties comprising high brightness (product of *ε* and *Φ*), a millisecond‐scale emission lifetime and high photostability, the EuL‐type materials demonstrated strong CPL with the magnitude of emission dissymmetry factors (*g*_lum_) peaking at around 0.3 [12, 13]. *g*_lum_ describes the net handedness of the emitted light and is expressed as

(1) $$g_{\mathrm{lum}}=\frac{2\left(I_{L}-I_{R}\right)}{\left(I_{L}+I_{R}\right)}$$

where *I*_L_ and *I*_R_ are the measured intensities of left‐handed CPL (LCPL) and right‐handed CPL (RCPL). This results in theoretically possible values of *g*_lum_ ranging between *g*_lum_ = 2 (100% LCPL) and *g*_lum_ = −2 (100% RCPL), whereas *g*_lum_ = 0 indicates no net circular polarization. Light is intrinsically circularly polarized because photons can take spin angular momentum values of +*h*/2π or −*h*/2π [14, 15, 16]. For the material to be considered CPL‐active, it needs to produce different amounts of LCPL and RCPL resulting in 0 < |*g*_lum_| < 2. The current highest *g*_lum_ value of 1.71 was recently recorded for an Eu^3+^ containing helicate (NEt_4_‐ΔΔ‐2) [17]. The previous *g*_lum_ record keeper was Cs[Eu(+)‐(hfbc)_4_] (hfbc = 3‐heptafluoro‐butylryl‐(+)‐camphorato) producing *g*_lum_ = 1.38 [18]. Although these values are significantly higher compared to those obtained from EuL‐type materials, other parameters such as low *Φ* (3%) and *ε* (35,000 M^−1^ cm^−1^) and absorption maximum at 310 nm would limit the practical application of Cs[Eu(+)‐(hfbc)_4_]. Although the *Φ* of 22% reported for NEt_4_‐ΔΔ‐2 was higher, other important parameters such as absorption maximum and *ε* were not reported.

One of the key limitations of CPL active materials is CPL sign fluctuation within the emission detection window, governed by transition dipole moments associated with the electronic states involved. Experimentally, simultaneous detection of LCPL and RCPL results in cancellation of the detected signal and therefore requires careful selection of band‐pass optical filters (BPFs) or monochromators targeting the wavelength regions containing single‐sign CPL bands. In addition, the selected CPL band must produce a high average *g*_lum_ value for a sufficient signal‐to‐noise ratio.

Currently, there is no reliable method of predicting the material's CPL spectral shape and *g*_lum_ based on the structure. Theoretically, *g*_lum_ is dependent on rotational and dipole strength, which results in the following relationship (Equation 2) between the *g*_lum_ and the magnitudes of the magnetic (*m*) and electric (*μ*) transition dipole moments as well as the cosine of the angle between their vectors (*θ*) [19, 20, 21]:

(2) $$\begin{array}{c} g_{\mathrm{lum}}=4\;\cos\;\theta \frac{\left|m\right|\left|\mu \right|}{m^{2}+\mu^{2}} \end{array}$$

In most photoluminescent molecular systems, *m* is significantly smaller compared to *μ*, which can simplify the equation:

(3) $$\begin{array}{c} g_{\mathrm{lum}}=4\;\cos\;\theta \frac{\left|m\right|}{\left|\mu \right|} \end{array}$$

As a result, the highest *g*_lum_ is achieved by minimizing the *μ* and maximizing the *m*, while also having the two vectors as parallel (or antiparallel) as possible. On the other hand, systems where absorption and emission of light are centered on the same structural entity, low magnitude of *μ* also results in low *ε*, suggesting a lower overall brightness. This is applicable to most organic chromophores that are designed to effectively absorb light via substantial displacement of electrical charge across the extended conjugated *π*‐networks upon photoexcitation, resulting in an inherently large *μ*. A similar occurrence happens in transition metal complexes where the *d* orbitals experience strong crystal field (CF), resulting in substantial relaxation of the Laporte selection rule that leads to a large *μ* and therefore a small *g*_lum_. On the contrary, the sensitized nature of emission in lanthanide(III) complexes allows maintaining high *ε*, while also minimizing *μ*. This is achieved by using the sensitizing chromophores for light absorption and lanthanide(III) center for light emission. In contrast to the transition metals, diffuse core‐like (shielded by 5 *s* and 5*p*) 4*f* orbitals experience CF weakly and therefore are essentially symmetry forbidden. As a result, most observed *f‐f* transitions are known as induced electric dipole transitions and are on a similar level of magnitude to the inherently weak magnetic dipole (MD) transitions [22]. Weak interaction between the 4*f* orbitals and the CF also contributes to the conservation of the MD moment that depends on spin and orbital angular momentum and therefore is sensitive to quenching by orbital mixing. In addition, lanthanides(III) possess strong spin‐orbit coupling, allowing for the spin contribution to the *f‐f* transitions, enhancing the associated MD moment (relaxes *ΔS* = 0 spin selection rule). The combination of all factors makes lanthanide(III) complexes, particularly those containing terbium(III) and europium(III), demonstrate the highest *g*_lum_ values (10^−1^‐10^0^) across other classes of CPL‐active materials, including chromium complexes (10^‐1^), helicenes (10^‐2^), ketones (10^‐2^), and cyclophanes (10^‐3^) [8, 23].

Accurate prediction of *g*_lum_ and CPL spectral profile is complex due to the strong dependence of *m* and *μ* transition dipole moments on the chiral environment surrounding the europium(III) center. EuL emission spectrum is dominated by the ^5^ *D*_0_ → ^7^ *F*_2_ (*ΔJ* = 2) transition that contains ~35% of the total emission [10]. The *ΔJ* = 2 transition is known as “hypersensitive” due to a strong dependence of its emission bands' splitting and relative intensity on the CF [24]. On the contrary, the ^5^ *D*_0_ → ^7^ *F*_1_ (*ΔJ* = 1) is essentially independent on the environment and often produces high *g*_lum_ due to its MD character. Simultaneously, *ΔJ* = 1 contributes only ~5% of the total emission, resulting in intensity trade‐off in real‐life application, for instance, CPL laser scanning confocal microscopy or CPL photography (CPLP) [9, 10, 13]. Therefore, it is desired to establish a predictable relationship between the structure of an EuL‐type material and its CPL properties, in particular, CPL sign conservation and average *g*_lum_ of the *ΔJ* = 2 emission manifold. This idea was explored in our previous work where a novel EuL complex containing three carboxylate donor groups resulted in a strong monosign CPL in *ΔJ* = 2 [10]. The present work investigates dependence of CPL on controlled structural modifications that are performed on different parts of the ligand. This is particularly important for the development of novel EuL‐type materials that can undergo a postmetal complexation‐stage functional group interconversion to facilitate a target‐based functionality. For example, such complex could be functionalized with glucose units for better cellular uptake advantageous in bioimaging [2]. Alternatively, it can be conjugated with various peptide sequences for targeting specific intracellular organelles such as endoplasmic reticulum with “AcCFFKDEL” or trans‐Golgi targeting “AcGASDYQRLGC” [25]. Such material could be also used for conjugation to chiral self‐assembly systems or chiral polymers for CPL enhancement [26, 27, 28]. Most importantly, to design a truly versatile material, its photophysical properties, including CPL spectral shape and *g*_lum_, must not change when the complex is functionalized. Therefore, the design of such EuL‐type material requires careful consideration of the impact of the proposed structural modifications on the europium(III) coordination environment and its symmetry.

## 3 Results and Discussion

### 3.1 Structural Specification of the Studied EuL^0^—EuL^4^ Materials

**FIGURE 2:**
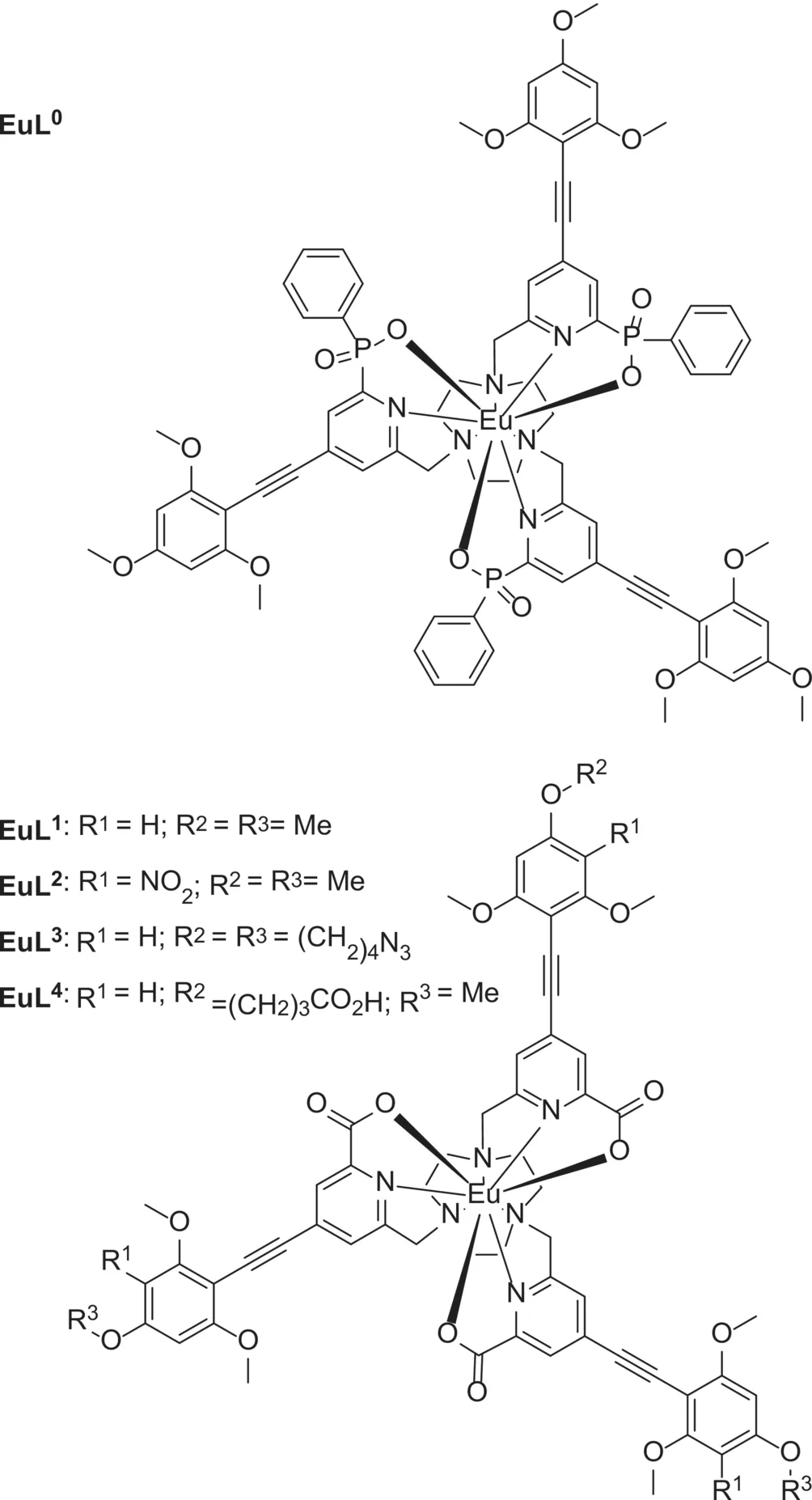
The structures of the previously reported **EuL** ^**0**^ and **EuL** ^**1**^ as well as novel **EuL** ^**2**^ **, EuL** ^**3**^ and **EuL** ^**4**^.

In this work, a series of EuL materials (Figure 2) was prepared where the structure was altered at different points with respect to the europium(III) center. The first two reference materials are the previously reported **EuL** ^**0**^ and **EuL** ^**1**^ complexes [10, 12]. They are EuL materials with 2,4,6‐trimethoxy substitution of the aryl ring for a red‐shifted ICT absorption band. The only structural difference between **EuL** ^**0**^ and **EuL** ^**1**^ is the nature of the donor group that is directly coordinated to the europium(III) center. The next investigated material, **EuL** ^**2**^, is a modification of **EuL** ^**1**^ where each of the three 2,4,6‐trimethoxy aryl rings was nitrated. Although such modification was spatially distant from the europium(III) coordination site, the nitro group was conjugated to the *π*‐network of the sensitizing chromophore, potentially impacting the photophysical properties of the whole complex. The next modification of **EuL** ^**1**^ to obtain **EuL** ^**3**^ was performed on a similar spatial separation from the europium(III) center as in **EuL** ^**2**^ but without affecting the original *π*‐network of the chromophore. This was achieved by replacing the para‐methoxy groups with azido butoxy moieties, which provided methoxy‐like electron‐donating properties to maintain the ~365‐nm absorption maximum. At the same time, the azides facilitated an effective sterically unhindered complex‐stage functionalization via “click‐chemistry” cycloaddition with alkyne‐bearing moieties under ambient reaction conditions, preventing complex decomposition or racemization. The **EuL** ^**0**^ to **EuL** ^**3**^ range could be assigned *quasi*‐*C*_3_ symmetry due to all three sensitizing chromophores being structurally identical, which was not the case for **EuL** ^**4**^. Although the modified structural part of **EuL** ^**4**^ was spatially separated from the europium(III) coordination site, the overall complex symmetry was lowered from trigonal to triclinic (*C*_1_). This was achieved by introducing structural inequivalence via modification of only one of the three chromophores with a butanoic acid group, whereas the other two chromophores were identical to that in **EuL** ^**1**^. In this example, a carboxylic acid was used instead of the azide to demonstrate the versatility of the modular EuL synthetic strategy. **EuL** ^**4**^ could be functionalized via amide bond formation with amine containing moieties. Because only one chromophore can be functionalized, **EuL** ^**4**^ is a good candidate for covalent association to larger structural entities such as nanoparticles or proteins, where **EuL** ^**3**^ could cause undesired aggregation due to a possibility of multiple functional group interconversion reactions per each complex molecule.

All complexes were prepared as racemic mixtures with enantiomers subsequently separated by chiral HPLC (see Supporting Information: Section 2.2) to analyze their CPL properties.

### 3.2 Synthetic Approaches Towards EuL^0^—EuL^4^ Materials

**SCHEME 1:**
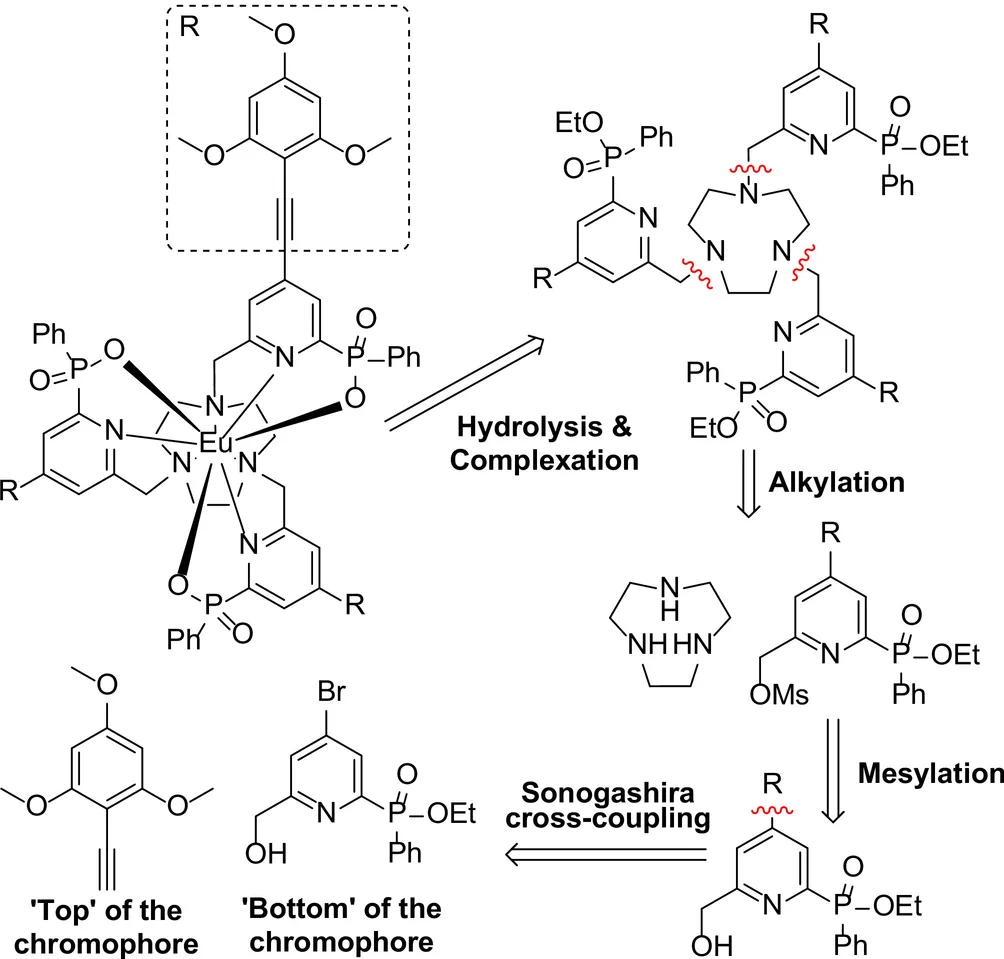
Retrosynthetic analysis of **EuL** ^**0**^.

**EuL** ^**0**^ and **EuL** ^**1**^ were synthesized using procedures adopted from literature and characterized (Supporting Information: Sections 1–5) [3, 6, 7, 29, 30]. The macrocyclic ligand must be prepared separately prior to hydrolysis of the esters required for complexation to europium(III) (Scheme 1).

**SCHEME 2:**
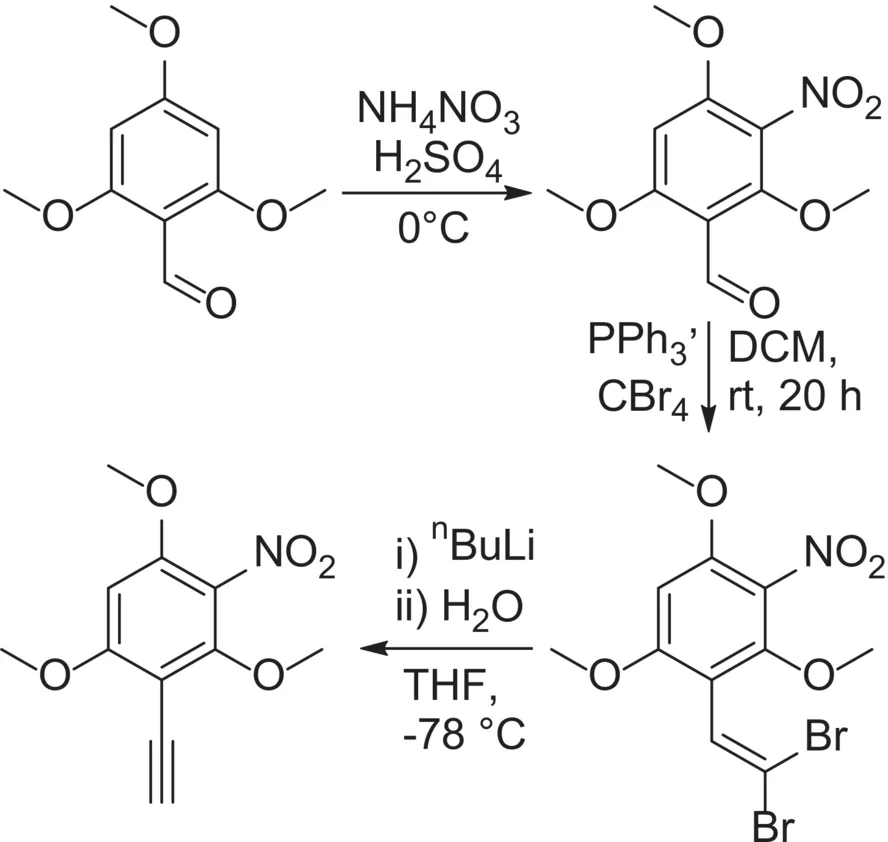
The reaction sequence for the “top” component of the chromophore in **EuL** ^**2**^.

The ligand is obtained by alkylation of TACN with mesylated chromophores that are obtained by Sonogashira cross coupling of two separately prepared “top” and “bottom” components (see Supporting Information: Sections 4 and 5). Although the “top” of the chromophore is shared between **EuL** ^**0**^ and **EuL** ^**1**^, the reaction sequence towards the phosphinate and carboxylate donor “bottom” components differs. Because **EuL** ^**2**^, **EuL** ^**3**^ and **EuL** ^**4**^ all contain carboxylate donor groups, they shared the “bottom” chromophore component with **EuL** ^**1**^. On the contrary, the “top” components were modified prior to completing the chromophore via cross coupling. In the case of **EuL** ^**2**^, the nitro group was introduced via nitration of a commercially available trimethoxy benzaldehyde (Scheme 2). Nitration was performed in the first synthetic step due incompatibility of acidic reaction conditions with later intermediates as well as relative inertness of the nitro group which remained unaffected throughout the rest of the synthetic procedure.

**SCHEME 3:**
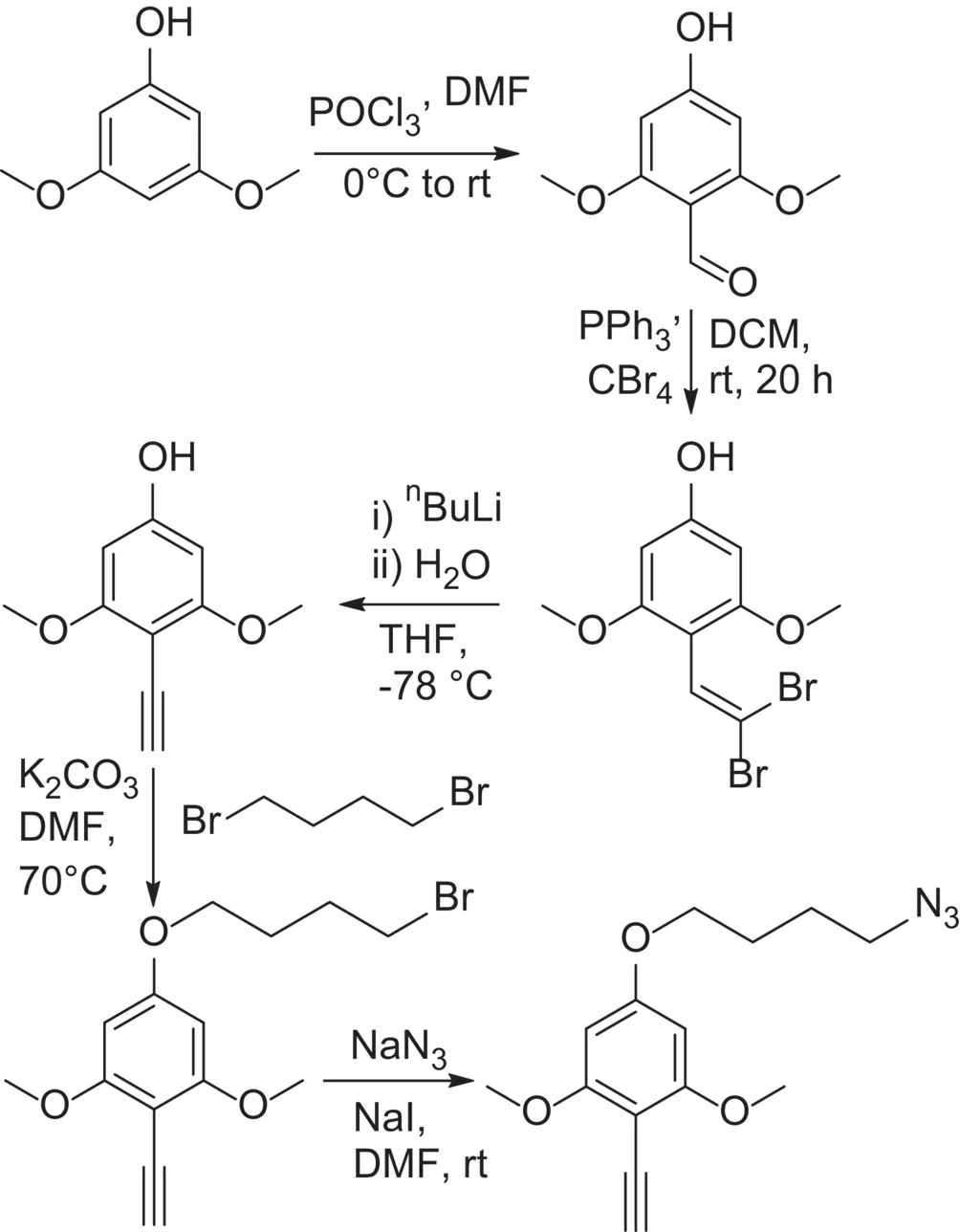
The reaction sequence for the “top” component of the chromophore in **EuL** ^**3**^.

A different strategy was implemented to prepare **EuL** ^**3**^, where the azide was introduced after the alkyne (Scheme 3). Such reaction sequence was chosen due to sensitivity of azide towards *n*‐butyllithium used in the second step of the Corey–Fuchs homologation reaction conditions.

The carboxylic acid containing “top” of the chromophore in **EuL** ^**4**^ was prepared following the same reaction sequence. The functionality was initially introduced as an ester to yield a carboxylic acid during the hydrolysis step prior to complexation.

**SCHEME 4:**
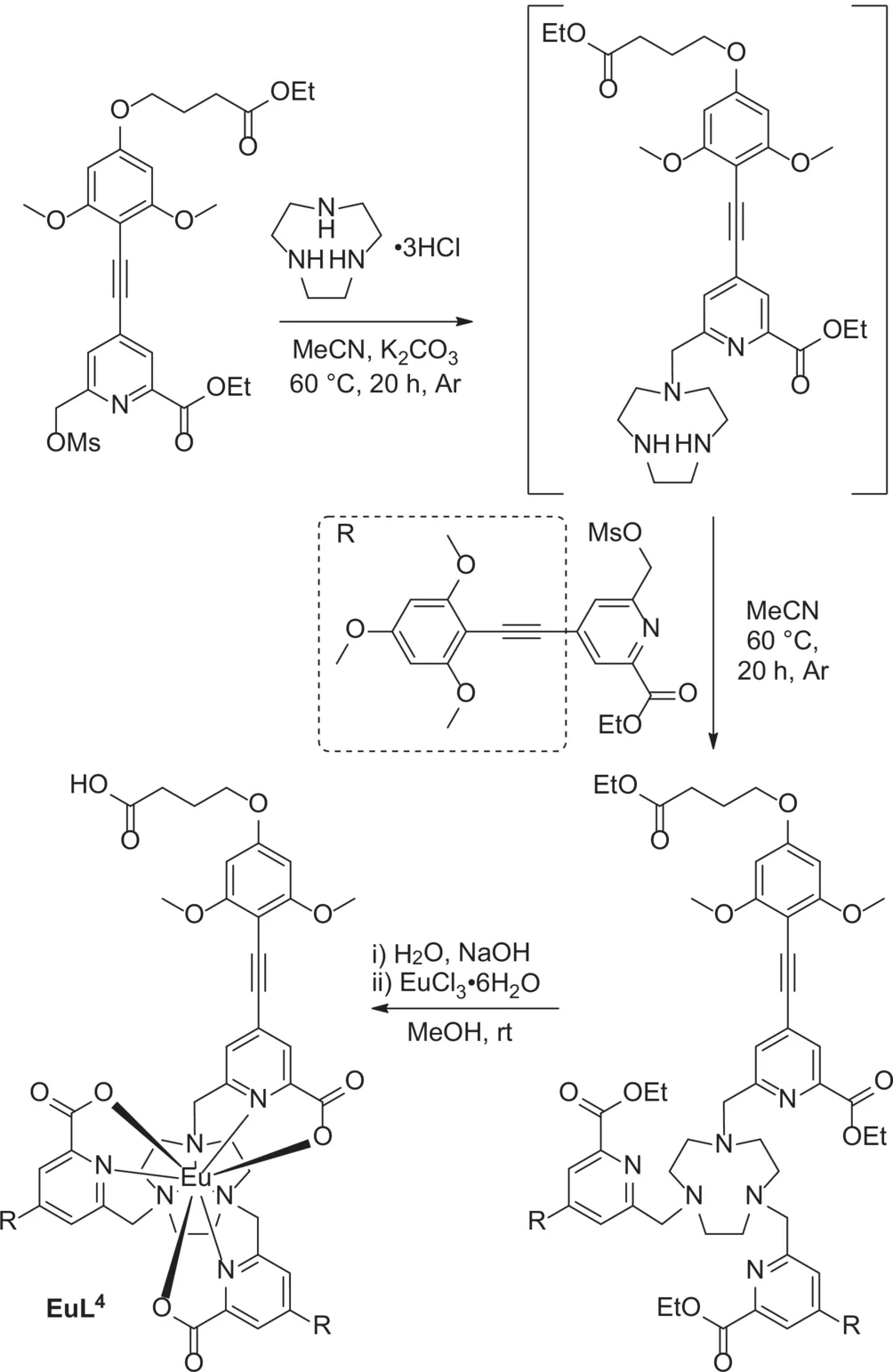
The reaction sequence for the sequential alkylation of the TACN with different chromophores, followed by hydrolysis and complexation to prepare **EuL** ^**4**^.

In this work, unsymmetric **EuL** ^**4**^ was prepared by sequential alkylation of TACN with different chromophores (Scheme 4) to maintain the simplicity of the method. Therefore, 1 equivalent of carboxylic acid‐functionalized chromophore was added first, followed by 2 equivalents of its unfunctionalized variant.

The standard strategy to yield unsymmetric EuL complexes was not suitable. It would involve attachment of structurally different chromophores to the TACN by preliminary protection of either one or two secondary amines of the TACN ring with *tert*‐butyloxycarbonyl (Boc) before alkylating with chromophores of one kind [31]. This is then followed by deprotection of the resulting species from Boc to attach chromophores of the other kind. Boc deprotection, however, requires a strong acid such as trifluoroacetic acid, which is incompatible with systems containing electron‐rich “top” component that makes the alkyne chemically potent. Electron‐rich groups at ortho and para positions relative to the alkyne provide resonance stabilization of the transition states of electrophilic addition of various species (e.g., water) to a protonated alkyne. The exposure of the alkyne to acidic conditions necessary for Boc deprotection could be avoided by modification of the synthetic procedure, where a doubly protected TACN is first alkylated with just the “bottom” bromopyridine component rather than a complete chromophore. Boc deprotection can be then performed to attach the rest of the chromophores. After that, the incomplete complex could be cross‐coupled to the carboxylic acid‐functionalized “top” of the chromophore.

### 3.3 Photophysical Characterization and Comparison of the EuL^0^—EuL^2^ Materials

**FIGURE 3:**
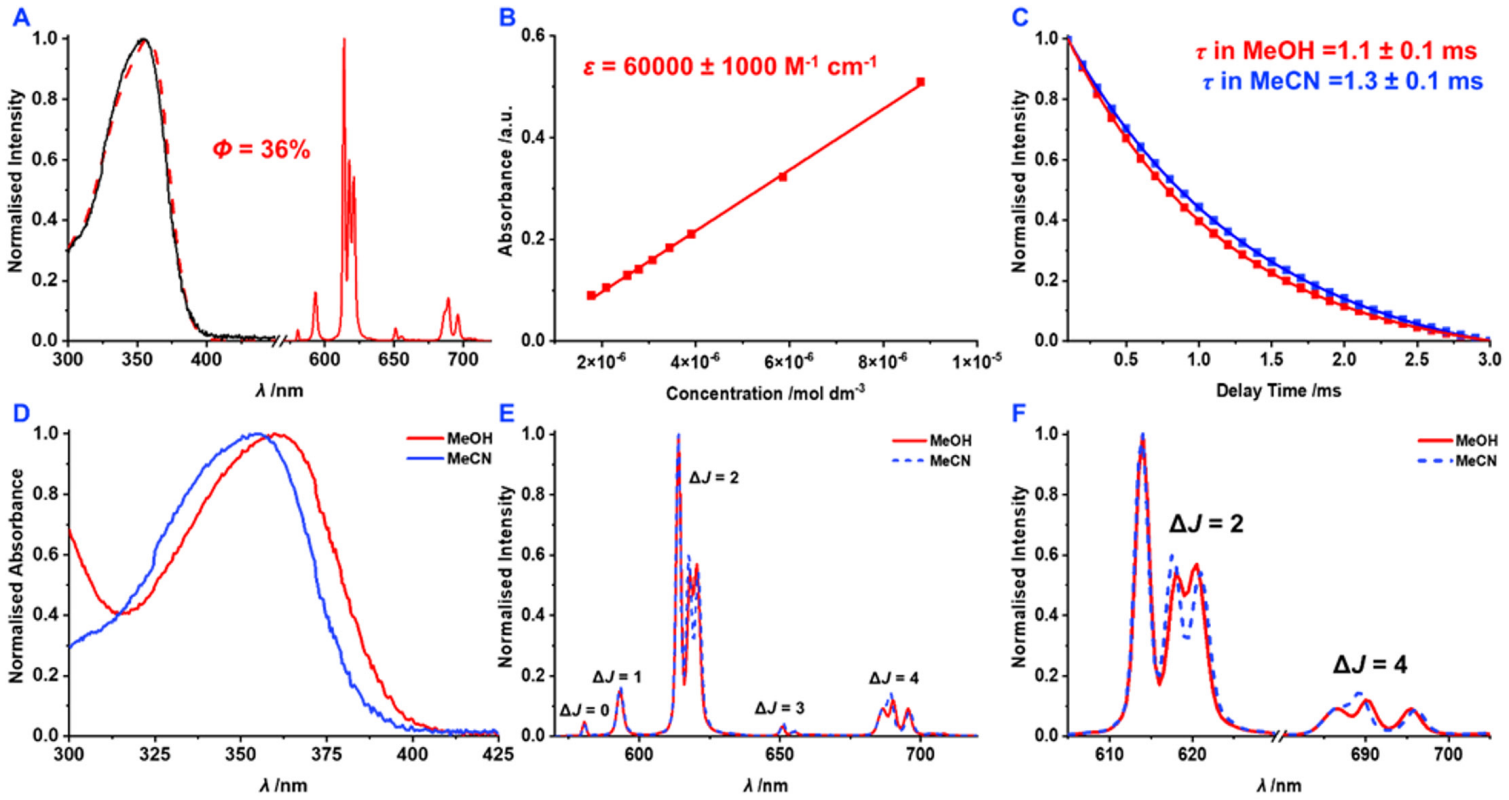
(A) Normalized absorption (black), emission (solid red, *λ*_exc_ = 360 nm) and excitation (dashed red, *λ*_em_ = 614 nm) spectra of **EuL** ^**0**^ in MeCN with the measured *Φ* (absolute method) annotated; (B) dependence of absorption (at 358 nm) on the **EuL** ^**0**^ concentration in MeCN; (C) lifetime plots of **EuL** ^**0**^ in MeOH and MeCN (0.1 abs solutions, *λ*_exc_ = 360 and 355 nm, *λ*_em_ = 614 nm); (D) normalized absorption spectra of **EuL** ^**0**^ in MeOH (red) and MeCN (blue); (E) normalized emission spectra of **EuL** ^**0**^ in MeOH (red, *λ*_exc_ = 360 nm) and MeCN (blue, *λ*_exc_ = 355 nm); (F) 605–630 and 680–720 nm sections of the spectrum in (E).

As expected from a sensitized europium(III) complex, a large pseudo‐Stokes shift of ~235 nm (from absorption maximum to *ΔJ* = 0) was observed (Figure 3A) between the ligand centered absorption band and europium(III) centered emission bands. As expected from a chromophore with electron‐rich 2,4,6‐trimethoxy aryl “top,” the absorption maximum was shifted towards the desired 365 nm (Figure 3A). In addition, the chromophore was susceptible to a positive solvatochromism, which was evidenced by a small ~5‐nm bathochromic shift of the absorption maximum when the solvent was changed from MeCN (*λ*_max_ = 355 nm) to MeOH (*λ*_max_ = 360 nm) (Figure 3D). The *ε* was measured (Figure 3B) in MeCN as 60,000 ± 1,000 M^−1^ cm^−1^ (at 360 nm), which was similar to other previously reported arylalkynylpyridine—sensitized europium(III) complexes [6, 32]. The *Φ* of **EuL** ^**0**^ was determined as 36% using an integrating sphere (see Supporting Information: Section 3.1). The measured *τ* of **EuL** ^**0**^ were 1.3 ± 0.1 ms in MeCN and 1.1 ± 0.1 ms in MeOH (Figure 3C), which agreed with the *τ* of related isostructural europium(III) complexes. A slightly shorter *τ* in MeOH, which is ICT‐stabilizing via H‐bonding, suggested a slight increase in the rate of BET from ^5^ *D*_0_ to ICT, which agreed with a decreased energy gap between the two states. In addition, the chromophore was susceptible to a positive solvatochromism, which was evidenced by a small ~5‐nm bathochromic shift of the absorption maximum when the solvent was changed from MeCN (*λ*max = 355 nm) to MeOH (*λ*max = 360 nm) (Figure 3D). As expected, the **EuL** ^**0**^ emission spectral profile (Figure 3E) comprised of europium(III) characteristic sharp bands with multiple ^5^ *D*_0_ → ^7^ *F*_J_ electronic transitions (Δ*J* = 0, 1, 2, 3, and 4) resolved. The presence of the low intensity Δ*J* = 0 transition agreed with the assigned *quasi‐C*_3_ symmetry, which also resulted in high intensity of the Δ*J* = 2. It demonstrated a splitting pattern containing a singlet and a doublet (each ~half intensity of the singlet), which corresponded to the *A* + 2*E* splitting of the ^7^ *F*_2_ (under *C*_3_ symmetry) [22, 24]. Dependence of the spectral profile on solvent was evident. For example, relative intensities of emission bands in Δ*J* = 2 and Δ*J* = 4 manifolds were different in MeCN and MeOH (Figure 3F).

**FIGURE 4:**
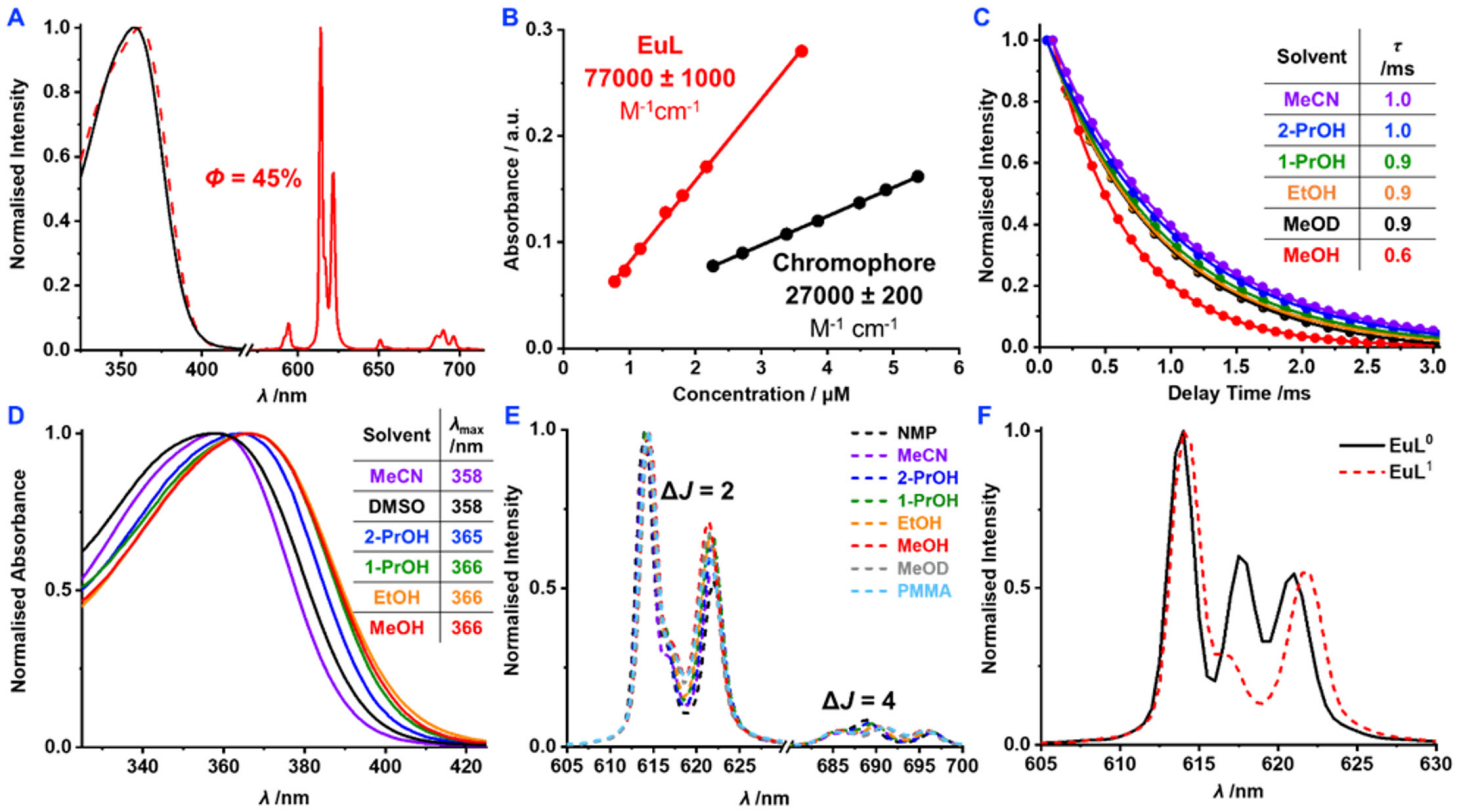
(A) Normalized absorption curves (black), emission (solid red, *λ*_exc_ = 360 nm) and excitation (dashed red, *λ*_em_ = 614 nm) spectra of **EuL** ^**1**^ in MeCN with the measured *Φ* (absolute method) annotated; (B) dependence of absorption (at 358 nm) on the **EuL** ^**1**^ concentration in MeCN; (C) lifetime plots of **EuL** ^**1**^ in different solvents (0.1 abs solutions, *λ*_em_ = 614 nm); (D) normalized absorption spectra of **EuL** ^**1**^ in different solvents; (E) normalized emission spectra (*Δ* *J* = 2 and 4 only) of **EuL** ^**1**^ in different solvents and solid PMMA film; (F) 605–630‐nm section of the spectrum in (E) demonstrating *Δ* *J* = 2 transition.

General photophysical properties of **EuL** ^**1**^ were mostly similar to those of **EuL** ^**0**^ (Summary in Table 1). It had a similar Stokes shift of ~235 nm (Figure 4A) and a characteristic appearance of the absorption and emission spectra that were centered on different parts of the sensitized system. *Φ* of **EuL** ^**1**^ was measured as 45% (in MeCN), which was 25% higher than that of **EuL** ^**0**^ (36% in MeCN). The *ε* of **EuL** ^**1**^ was determined as 77,000 ± 1000 M^−1^ cm^−1^ (*λ*_exc_ = 358 nm, MeCN) (Figure 4B), which was ~28% higher than 60,000 ± 1000 M^−1^ cm^−1^ (*λ*_exc_ = 355 nm, MeCN) recorded for **EuL** ^**0**^. In order to confirm the accuracy of the determined *ε*, the experiment was repeated with a single chromophore to produce 27,000 ± 200 M^−1^ cm^−1^ (*λ*_exc_ = 340 nm) in MeCN, which, as expected, was approximately one‐third of that measured for **EuL** ^**1**^ containing three chromophores. As a result, **EuL** ^**1**^ was found to be a significantly brighter material compared to **EuL** ^**0**^, because both *Φ* and *ε* were higher. The brightness (*B*), which is a product of *Φ* and *ε*, was calculated as 34,650 M^−1^ cm^−1^ (*λ*_exc_ = 358 nm) for **EuL** ^**1**^, and 21,600 M^−1^ cm^−1^ (*λ*_exc_ = 355 nm) for **EuL** ^**0**^, resulting in an overall ~60% increase attributed to the replacement of the chromophore's phenylphosphinate donor group.

**TABLE 1:** Comparison of the key photophysical properties and racemization of **EuL** ^**0**^ and **EuL** ^**1**^ in MeCN.

Parameter	EuL^0^	EuL^1^	% increase
*λ*_max_/nm	356	358	**—**
*ε*/M^−1^ cm^−1^	60,000	77,000	**28%**
*Φ*	0.36	0.45	**25%**
*B/*M^−1^ cm^−1^	22,000	34,650	**58%**
*τ*/ms	1.3 ± 0.1	1.0 ± 0.1	**−30%**
*σ* ^2^/GM	132 (at 720 nm)	308 (at 700 nm)	**133%**
max|*g*_lum_| *ΔJ* = 1	0.21	0.30	**43%**
max|*g*_lum_ *ΔJ* = 2	0.028	0.071	**154%**
* *CPB*_*i*_ *ΔJ* = 1	166	307	**85%**

* Circularly polarized brightness for an individual transition (see Supporting Information: Section 3.3).

**EuL** ^**1**^ demonstrated a significantly shorter *τ* of **EuL** ^**1**^ in MeOH (0.6 ms) compared to other solvents, where *τ* remained at a constant ~1 ms in solvents with different polarity as well as in monodeuterated MeOH (MeOD) (Figure 4C). Because *τ* in MeOD was similar to that in the rest of the alcohols, it was supposed that emission quenching is not a product of vibrational relaxation via O‐H oscillators. Instead, small protic solvents could stabilize the ICT via H‐bonding, which decreased the energy gap between the ICT state and the lower lying ^5^ *D*_0_, increasing the rate of thermally activated back energy transfer (BET). This effect was not as significant in **EuL** ^**0**^ as bulky phenyl groups could sterically hinder the H‐bond accepting pyridyl nitrogen from forming linear hydrogen bonds with the solvent. Stabilization of the ICT state could also be evidenced by the positive solvatochromism observed from the **EuL** ^**1**^ absorption spectra in solvents with increasing polarity and H‐bond donating properties (Figure 4D). Importantly, no significant dependence of the **EuL** ^**1**^ emission spectral shape on the solvent was observed (Figure 4E). The emission spectral profile remained the same, while the relative intensity of the emission bands in the *ΔJ* = 2 manifold slightly changed in different solvents.

This was not the case for **EuL** ^**0**^, where a slight change in the band splitting was observed in **EuL** ^**0**^ within the *ΔJ* = 2 and 4 manifolds when the solvent was changed (Figure 4F). Similarly to **EuL** ^**0**^, the environmentally “hypersensitive” *ΔJ* = 2 emission manifold of **EuL** ^**1**^ appeared as three bands (Figure 4F).

**FIGURE 5:**
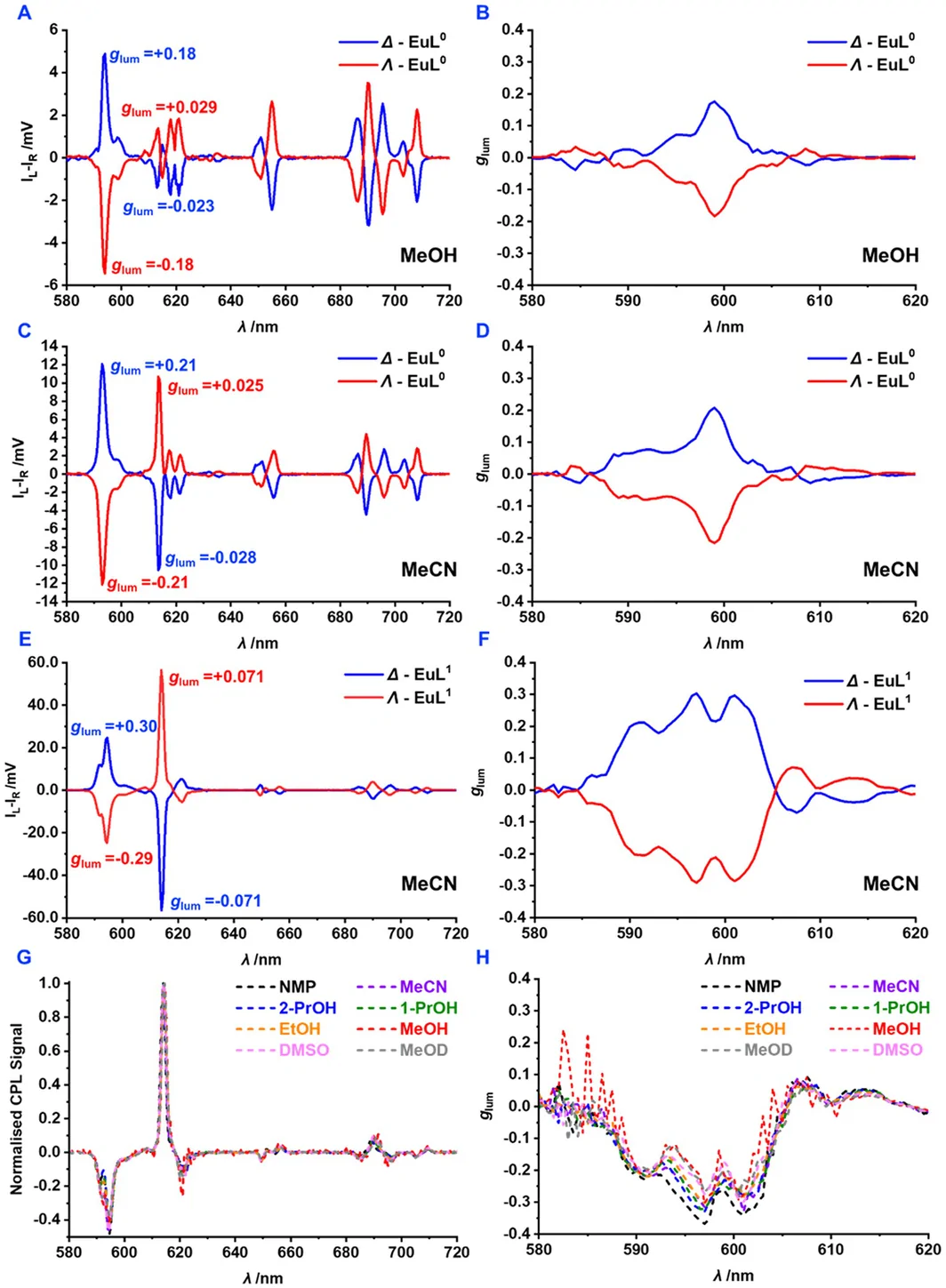
CPL spectra of ***Δ*** (blue) and ***Λ*** (red) enantiomers of **EuL** ^**0**^ in MeOH (*λ*_exc_ = 365 nm, 5 avg.) (A) and MeCN (*λ*_exc_ = 360 nm, 5 avg.) (C) and of **EuL** ^**1**^ in MeCN (*λ*_exc_ = 365 nm, 5 avg.) (E) (with peak *g*_lum_ values stated) and the corresponding *g*_lum_ plots (B, D, and F); normalized (to the intensity at 614 nm) CPL spectra of ***Λ***‐**EuL** ^**1**^ in different solvents (*λ*_exc_ = 365 nm, 5 avg.) (G) and the corresponding *g*_lum_ plot (H).

The effect of the nature of the chromophores' donor groups that are directly coordinated to the europium(III) center on the electronic structure was further studied by analyzing the CPL spectra and ***g_lum_*** plots (Figure 5A–H) of *Δ* and *Λ* enantiomers **EuL** ^**0**^ and **EuL** ^**1**^. The enantiomers were assigned based on the CPL spectral fingerprint of the isostructural materials, the enantiomers of which were assigned using single‐crystal x‐ray diffraction in previous studies [30, 33].

Comparison of **EuL** ^**0**^ CPL spectral shape in MeOH (Figure 5A) and MeCN (Figure 5C) demonstrated a significant difference in the appearance of the *ΔJ* = 2 manifold, whereas the rest of the observed transitions remained the same. *ΔJ* = 2 comprised three emission bands that corresponded to the transitions from ^5^ *D*_0_ to 3 Stark states (*A* + 2*E)* of ^7^ *F*_2_ that are no longer degenerate due to the CF of the *C*_3_ symmetry. In MeOH, however, four bands could be distinguished, suggesting additional splitting of states that could be a result of the phosphinate donor oxygen behaving as H‐bond acceptor in protic solvents.

The main consequence of replacing phenylphosphinate donor groups with carboxylate groups is substantial increase of *ΔJ* = 2 CPL intensity (Figure 5E), making it higher compared to *ΔJ* = 1, which is normally the most CPL active due to its MD character. This could be a consequence of a more symmetric europium(III) environment, suggested by a weaker splitting of bands in the *ΔJ* = 2 manifold. This enhanced the conservation of *g*_lum_ sign and magnitude over wider wavelength ranges in **EuL** ^**1**^. Although the magnitude of the maximal *g*_lum_ of *ΔJ* = 2 (0.071 at 607 nm) was still lower than that of *ΔJ* = 1 (0.30 at 597 nm), the difference in brightness of *ΔJ* = 1 and *ΔJ* = 2 transitions resulted in their similar practical value due to the associated signal‐to noise ratios [10]. In other words, the strong CPL intensity of *ΔJ* = 2 compensated for its lower *g*_lum_ when compared to *ΔJ* = 1 and vice versa. This was not the case for **EuL** ^**0**^ where only *ΔJ* = 1 was practically useful. More specifically, **EuL** ^**0**^ in MeCN produced maximal *g*_lum_ values with magnitudes of 0.21 (at 599 nm) and 0.028 (at 609 nm) for *ΔJ* = 1 and *ΔJ* = 2, respectively (Figure 5D). As a result, **EuL** ^**1**^ produced *ΔJ* = 1 and *ΔJ* = 2 maximal *g*_lum_ values that were 43% and 154% higher in magnitude than those of **EuL** ^**0**^. When average *g*_lum_ magnitude values for the single‐sign regions of *ΔJ* = 1 and *ΔJ* = 2 manifolds were compared, **EuL** ^**1**^ produced 0.18 (*ΔJ* = 1, 585–605 nm) and 0.023 (*ΔJ* = 2, 610–618 nm) (Figure 5F) that were 137% and 130% higher than 0.076 (*ΔJ* = 1, 585–605 nm) and 0.010 (*ΔJ* = 2, 610–618 nm) produced by **EuL** ^**0**^ (Figure 5D). Such increase in *g*_lum_ achieved by a replacement of chiral phosphinate donors with achiral bidentate carboxylate donors increased the MD moment (total angular momentum driven), affecting the overall rotatory strength of the molecule and the variation of CPL sign within the *ΔJ* = 1 and *ΔJ* = 2 manifolds. An increase in rotational strength improved emission dissymmetry, leading to higher values of *g*_lum_. In contrast to the pseudotetrahedral geometry of phosphinate donors in **EuL** ^**0**^, trigonal planar geometry of carboxylate donors in **EuL** ^**1**^ placed the coordinating oxygen atoms further from the europium(III) center. This led to a weaker CF experienced by europium(III), which reduced the splitting of individual ^7^ *F*_*J*_ electronic states, which was particularly significant for the spectral shape of the “hypersensitive” *ΔJ* = 2 transition manifold.

Another important consequence of using the carboxylate donor is independence of total emission and CPL spectral profile on solvent (Figure 5G). This could be attributed to a bidentate coordination of the symmetrical carboxylate oxygens that share equally distributed negative charge, resulting in a stronger interaction with europium(III) and therefore weaker interaction with the solvent molecules. The observed *g*_lum_ variation in different solvents was also insignificant (Figure 5H). Small variation in *g*_lum_ could be mostly related to a change in signal‐to‐noise ratio governed by the total emission intensity rather than an extrinsic solvent effect.

**FIGURE 6:**
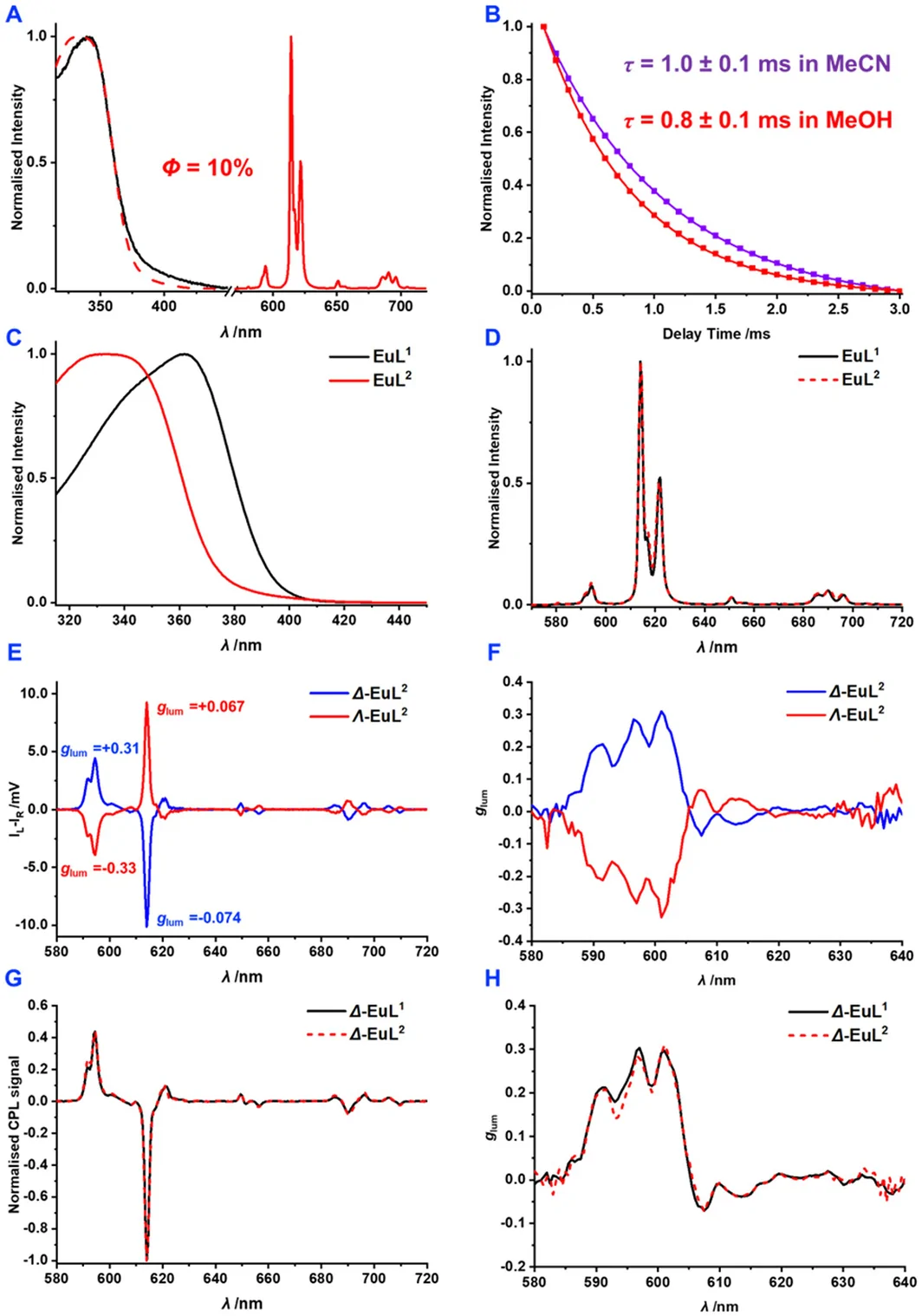
(A) Normalized absorbance (black), emission (solid red, *λ*_exc_ = 340 nm) and excitation (dashed red, *λ*_em_ = 614 nm) spectra of **EuL** ^**2**^ in MeCN with the measured *Φ* (absolute method) annotated; (B) lifetime plots of **EuL** ^**2**^ in MeCN and MeOH (0.1 abs solutions, *λ*_exc_ = 340 nm, *λ*_em_ = 614 nm); (C) normalized excitation spectra of **EuL** ^**1**^ (black) and **EuL** ^**2**^ (red) in MeCN (λ_em_ = 614 nm); (D) normalized emission spectra of **EuL** ^**1**^ (black) and **EuL** ^**2**^ (dashed red) in MeCN (*λ*_exc_ = 360 and 340 nm, respectively). (E) CPL spectra of ***Δ***‐**EuL** ^**2**^ (blue) and ***Λ***‐**EuL** ^**2**^ (red) in MeCN (*λ*_exc_ = 340 nm, 5 averages); (F) g_lum_ plots for the CPL spectra in (E) (*Δ* *J* = 1 and 2 only); (G) normalized (to the intensity at 614 nm) CPL spectra of the ***Δ*** enantiomers of **EuL** ^**1**^ (solid black) and **EuL** ^**2**^ (dashed red) in MeCN (*λ*_exc_ = 360 nm for **EuL** ^**1**^ and 340 nm for **EuL** ^**2**^, 5 averages); (H) *g*_lum_ plots for the CPL spectra in **G** (*Δ* *J* = 1 and 2 only).

Since the rest of the **EuL** ^**2**^‐**EuL** ^**4**^ series contained carboxylate donor groups, their photophysical properties were directly compared to **EuL** ^**1**^ rather than **EuL** ^**0**^. As mentioned previously, **EuL** ^**2**^ is a modification of **EuL** ^**1**^, where each chromophore was substituted with a nitro group at the trimethoxy aryl “top.” Therefore, it was directly bonded to the conjugated *π*‐network, affecting the electronic structure of the sensitizing system, which affected the photophysical properties (Figure 6A–H). As a result, the absorption maximum of **EuL** ^**2**^ in MeCN was recorded at ~340 nm, suggesting ~20‐nm hypsochromic (blue) shift from that of **EuL** ^**1**^ (Figure 6C). This is a result of the electron withdrawing nature of a nitro group that decreased the energy of chromophores' HOMO.

*Φ* of **EuL** ^**2**^ (10%) was around four times lower than that of **EuL** ^**1**^ (45%). On the other hand, the emission lifetime of **EuL** ^**2**^ in MeCN remained the same with that of **EuL** ^**1**^ (both 1.0 ± 0.1 ms) (Figure 6B). Combination of these factors suggested that the emission of **EuL** ^**2**^ was quenched by a nonradiative relaxation of the ICT excited state of the chromophore rather than europium(III) ^5^ *D*_0_ long‐lived state. From previous *τ* experiments, it was evident that *τ* of the parent **EuL** ^**1**^ significantly decreased in MeOH (but not in MeOD). The quenching was therefore attributed to ICT state stabilization via H‐bonding, promoting the thermally activated BET. In this study of **EuL** ^**2**^, *τ* in MeOH was recorded as 0.8 ± 0.1 ms (Figure 6B), which was ~33% higher compared to 0.6 ± 0.1 ms for **EuL** ^**1**^. Although ICT was still stabilized by H‐bonding, the electron withdrawing nitro groups had a destabilizing effect. Although the mechanism of such ICT state destabilization was not determined, it was hypothesized that the ICT state could exist in a form of charge separated species, where a negative charge localized on the electron‐depleted in the ground state pyridyl nitrogen, resulting in a positive charge on the electron‐pushing aryl group. In the case of **EuL** ^**2**^, such positive charge was proximal to an electron withdrawing nitro group, giving rise to an unfavorable electrostatic interaction.

There was no significant difference between the total intensity (Figure 6A,D), CPL spectra (Figure 6E,G) and *g*_lum_ plots (Figure 6F,H) of the enantiomers of **EuL** ^**2**^ and those of **EuL** ^**1**^. It was particularly important that even the “hypersensitive” *ΔJ = 2* emission manifolds of the two materials did not produce a significant difference in spectral shape and relative intensity of the bands (Figure 6G). The maximum *g*_lum_ was recorded as +0.31 (at 601 nm) for ***Δ*‐EuL** ^**2**^ and −0.32 (at 601 nm) for ***Λ*‐EuL**_**2**_ that were similar to +0.30 and −0.29 for the enantiomers of **EuL** ^**1**^ (at 597 nm). The highest *g*_lum_ values recorded for the *ΔJ = 2* were −0.074 and +0.067 (both at 607.5 nm) for the ***Δ*‐** and ***Λ*‐EuL** ^**2**^, respectively, which were similar to ±0.071 for **EuL** ^**1**^ (at 607 nm) (Figure 6H).

### 3.4 Complex‐Stage Functionalization and Photophysical Characterization of the EuL^3^ and EuL^3g^ Materials

**SCHEME 5:**
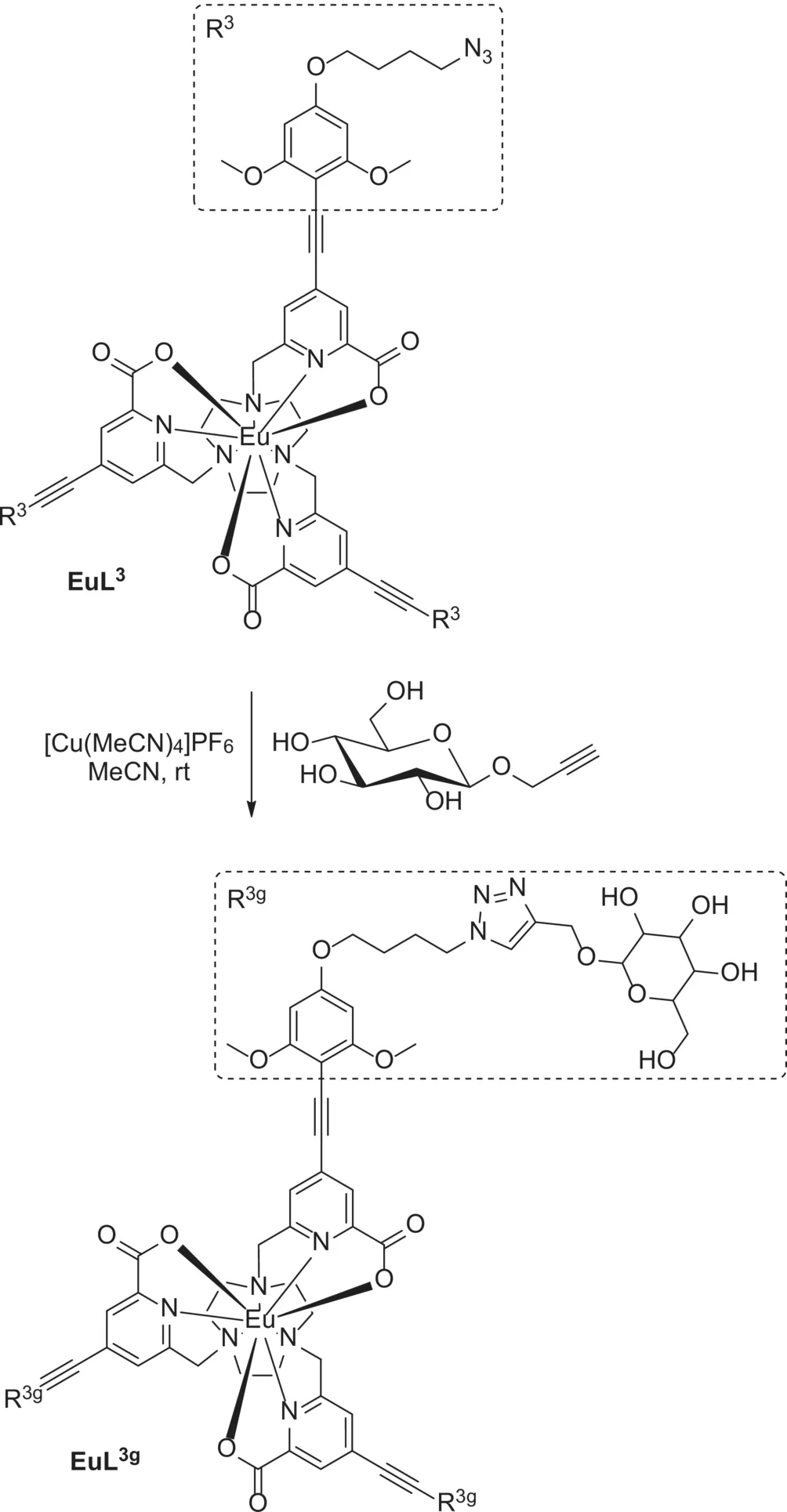
The reaction sequence for the proof‐of‐concept complex‐stage functionalization of **EuL** ^**3**^ with propargyl glucopyranoside.

**FIGURE 7:**
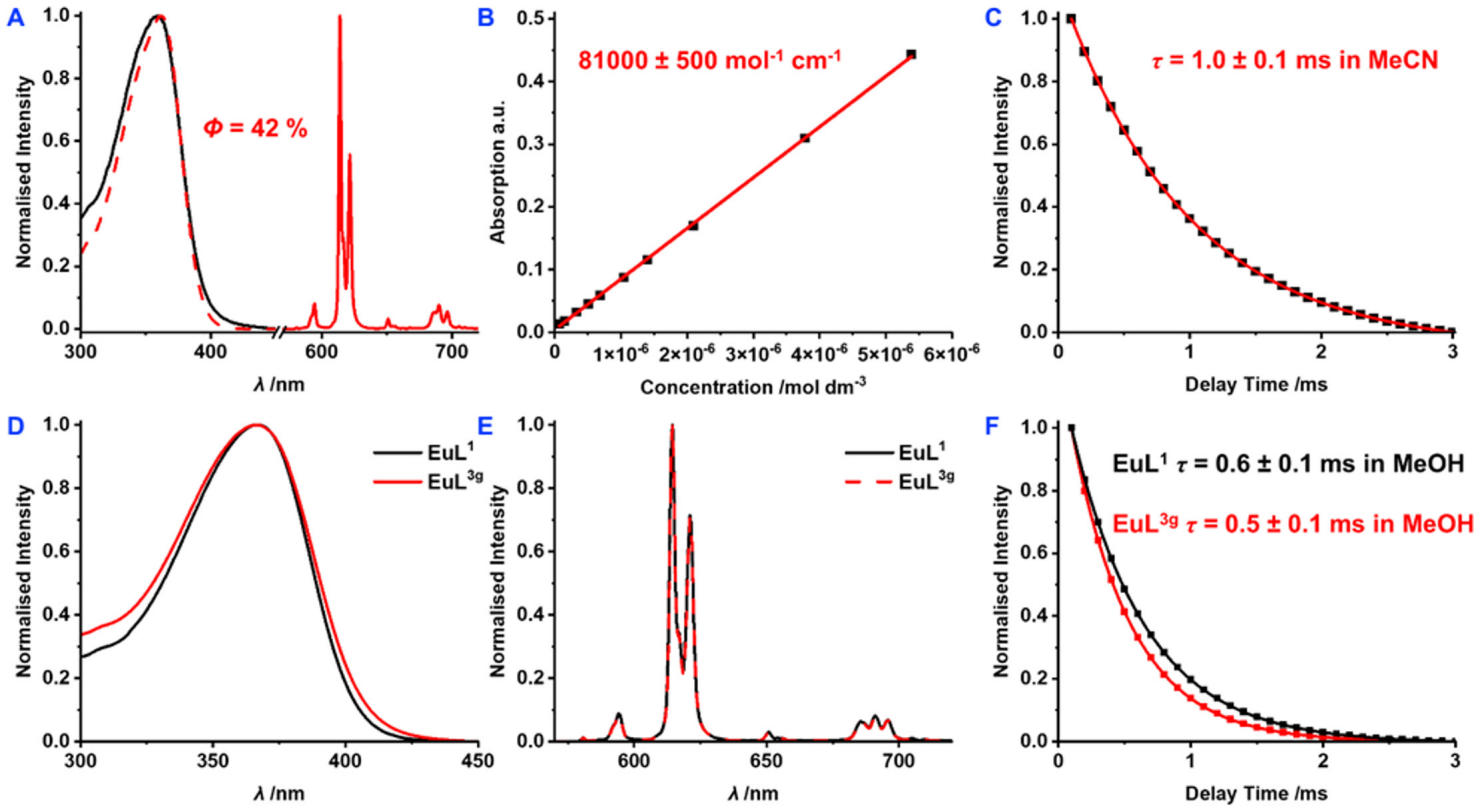
(A) Normalized absorption (black), emission (solid red, *λ*_exc_ = 360 nm) and excitation (dashed red, *λ*_em_ = 614 nm) spectra of **EuL** ^**3**^ in MeCN with the measured *Φ* (absolute method) annotated; (B) dependence of absorption on the **EuL** ^**3**^ concentration in MeCN (C) lifetime plot of **EuL** ^**3**^ in MeCN (0.1 abs solutions, *λ*_exc_ = 360 nm, *λ*_em_ = 614 nm); (D) normalized excitation spectra of **EuL** ^**1**^ (black) and **EuL** ^**3g**^ (red) in MeOH (*λ*_em_ = 614 nm); (E) normalized emission spectra of **EuL** ^**1**^ (black) and **EuL** ^**3g**^ (dashed red) in MeOH (*λ*_exc_ = 365 nm); (F) lifetime plots of **EuL** ^**1**^ (black) and **EuL** ^**3g**^ (red) in MeOH (0.1 abs solutions, *λ*_exc_ = 365 nm, *λ*_em_ = 614 nm).

This investigation of photophysical properties of **EuL** ^**2**^ evidenced how structural modifications on the far end from the europium(III) center did not result in perturbation of the local symmetry that would affect the CF splitting of the ^7^ *F*_*J*_ states, and, as a result, the CPL spectral band shape and relative intensity remained unaffected. On the other hand, due to direct conjugation of the nitro group with the *π*‐network of the chromophore, other sensitizer‐related parameters such as *Φ*, absorption maximum and lifetime were altered and obtained environmental dependence. As a result, such **EuL** ^**2**^‐type materials could be further explored as environment‐responsive probes with europium(III) emission‐based internal references, for example, relative intensity of *ΔJ* = 1 and *ΔJ* = 2 emission bands and *g*_lum_. On the contrary, **EuL** ^**2**^‐type modification is not a perfect candidate for a versatile material with fixed photophysical properties that can be functionalized at the complex‐stage. Firstly, functionalization of the nitro group would be challenging due to steric hindrance from the adjacent methoxy groups. Secondly, complex stage functionalization at the aryl ring of the chromophore would alter the photophysical properties, which would reduce the material's versatility and require further application‐specific optimizations. Therefore, **EuL** ^**3**^ was designed to address these limitations by introducing a “click‐chemistry” compatible azide that was separated from the rest of the complex by an alkyl chain. This was successfully demonstrated by reacting **EuL** ^**3**^ with a commercially available D‐glucose derivative containing an alkyne group under click‐chemistry conditions (Scheme 5). The absorption and emission profiles of the **EuL** ^**3**^ in MeCN matched those of the **EuL** ^**1**^ (Figure 7A). The molar extinction coefficient (Figure 7B) recorded for **EuL^3^** (81,000 ± 500 mol^−1^ cm^−1^) was slightly higher than that of **EuL^1^** (77,000 ± 500 mol‐1 cm‐1), which could be due to a butoxy group being slightly more electron‐donating than methoxy, providing a slightly stronger electric transition dipole. The absorption maxima (~360 nm, MeCN) matched due to conservation of the chromophores' electronic structure, and hence, the HOMO‐LUMO energy gap provided by a similar electron‐donating effect of the methoxy (in **EuL** ^**1**^) and butoxy (in **EuL** ^**3**^ and **EuL** ^**3g**^) substituents at the para position of the aryl ring. This also agrees with similar values of *Φ* (45% in **EuL** ^**1**^ and 42% in **EuL** ^**3**^) and *τ* (both 1.0 ± 0.1 ms) for both materials (Figure 7C). The matching emission band shapes (Figure 7A) agreed with the equivalent local symmetry at the europium(III) center provided by the two ligands.

The photophysical measurements for the glucose‐functionalized **EuL** ^**3g**^ were performed in MeOH because the material was practically insoluble in MeCN. The data for **EuL** ^**3g**^ was, again, compared to the reference **EuL** ^**1**^ to confirm that the complex‐stage functionalization of the **EuL** ^**3**^ did not alter the desired photophysical properties. As expected, both emission (Figure 7D) and absorption (Figure 7E) band shapes matched, where the absorption maximum of **EuL** ^**3g**^ in MeOH was found at 365 nm, matching to that of **EuL** ^**1**^. In addition, both materials produced nearly matching emission lifetimes (Figure 7F). Although the enantiomers of **EuL** ^**3**^ were successfully separated using analytical‐scale chiral HPLC (see Figure S2.5), CPL spectra of **EuL** ^**3**^ and **EuL** ^**3g**^ were not recorded. **EuL** ^**3**^ was obtained by modification of **EuL** ^**1**^ that was performed even further from the europium(III) coordination environment than in the case of **EuL** ^**2**^. Because functionalization of the **EuL** ^**2**^ chromophore did not produce any changes in the CPL spectra and the *g*_lum_ plots, which were equivalent to those of **EuL** ^**1**^, high probability of the conservation of the CPL properties of **EuL** ^**3**^ and **EuL** ^**3g**^ was also expected. So far, the modified **EuL** ^**2**^ and **EuL** ^**3**^ complexes remained of *quasi*‐*C*_3_ symmetry overall due to the three chromophores being structurally identical. Equivalent chromophores can be a disadvantage in scenarios where complex functionalization with different groups is desired. Such design also imposes limitations on selective attachment of complex molecules to other moieties, for example, chiral macrocycles, nanoparticles, or even viruses, where simultaneous attachment via two or three functionalized chromophores could result in complex geometry distortion or cause undesired aggregation.

**FIGURE 8:**
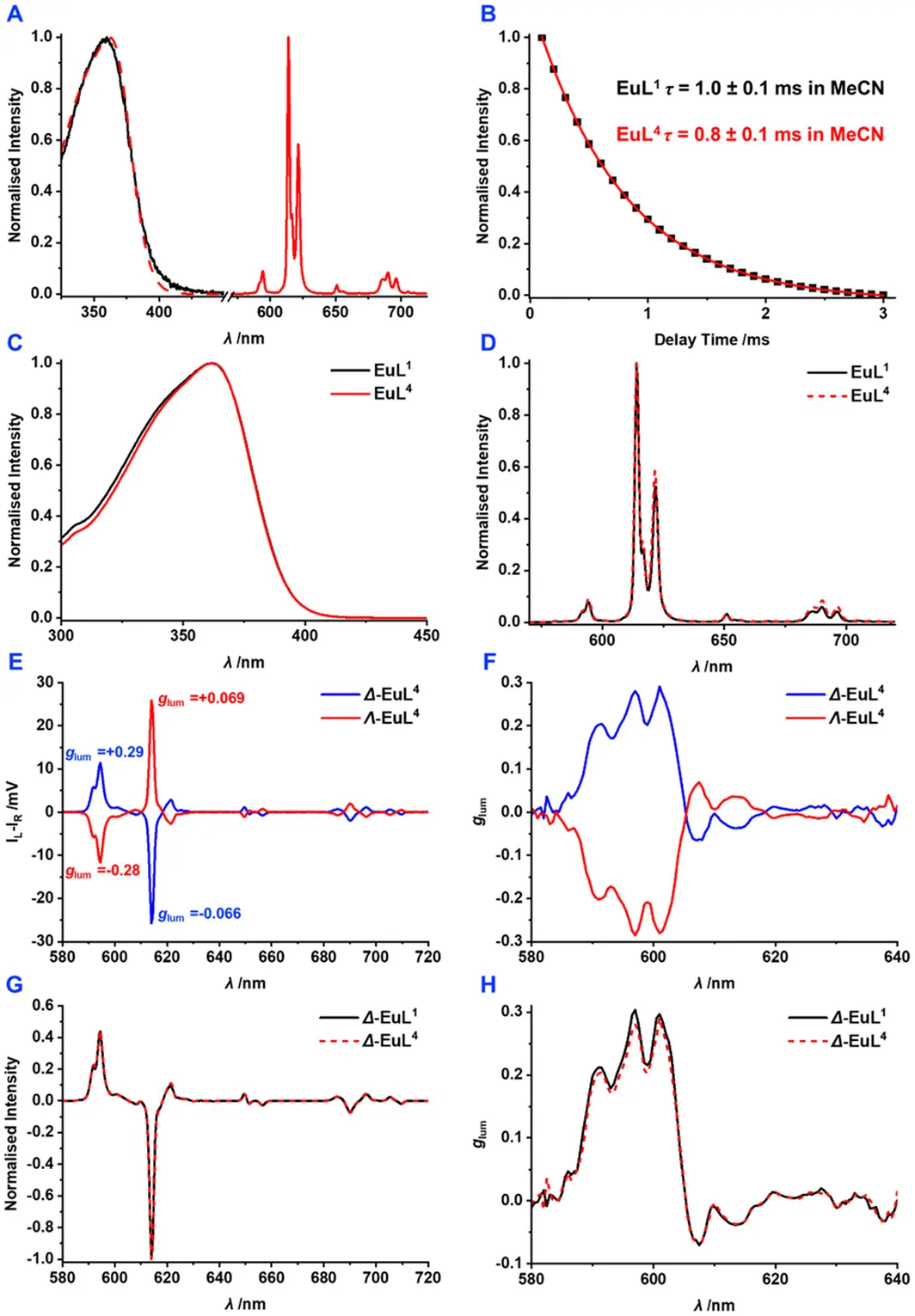
(A) Normalized absorption (black), emission (solid red, *λ*_exc_ = 360 nm) and excitation (dashed red, *λ*_em_ = 614 nm) spectra of **EuL** ^**4**^ in MeCN; (B) emission lifetime plot (0.1 abs solutions, *λ*_exc_ = 360 nm, *λ*_em_ = 614 nm) of **EuL** ^**4**^ in MeCN; (C) normalized excitation spectra (*λ*_em_ = 614 nm) of **EuL** ^**1**^ (black) and EuL^4^ (red) in MeCN; (D) normalized emission spectra (*λ*_exc_ = 360 nm) of **EuL** ^**1**^ (solid black) and **EuL** ^**4**^ (dashed red) in MeCN; (E) CPL spectra of the ***Δ***‐**EuL** ^**4**^ (blue) and ***Λ**‐* **EuL** ^**4**^ (red) in MeCN (λ_exc_ = 360 nm, 5 averages); (F) *g*_lum_ plots for the CPL spectra in (E) (*ΔJ* = 1 and 2 only); (G) normalized (to the intensity at 614 nm) CPL spectra of the ***Δ*** enantiomers of **EuL** ^**1**^ (solid black) and **EuL** ^**4**^ (dashed red) in MeCN (*λ*_exc_ = 360 nm, 5 averages); (H) *g*_lum_ plots for the CPL spectra in **C** (*ΔJ* = 1 and 2 only).

The overall symmetry of **EuL** ^**4**^ can be assigned as *C*_1_. In theory, if europium(III) is placed in a coordination environment with triclinic symmetry, it would induce additional band splitting, resulting in three Stark states of ^7^ *F*_1_ and five states of ^7^ *F*_2_, resulting in three and five emission bands observed in *ΔJ* = 1 and *ΔJ* = 2 manifolds, respectively. As evidenced by the photophysical study of **EuL** ^**0**^, **EuL** ^**1**^, and **EuL** ^**2**^, only the coordination environment of europium(III) center affected the emission spectra. Therefore, as expected, the photophysical properties of **EuL** ^**4**^ matched with **EuL** ^**1**^ suggesting no detectable effect of the overall molecular symmetry reduction via distant structural modifications on the energy and splitting of europium(III) electronic states (Figure 8A–H).

In order to estimate whether an implemented structural modification would affect the CPL spectral shape, the relative intensity of the emission bands and *g*_lum_, the rotational strength of a molecule, governed by *μ* and *m* transition dipole moments need to be considered. In sensitized lanthanide complexes, where absorption and emission processes are centered at different parts of the molecule, only transition dipole moments associated with the emitting lanthanide center affected CPL. Because the coordination environment of europium(III) center remained unaltered by the implemented modification, the CF splitting of the ^7^ *F*_*J*_ electronic states and, therefore, the CPL spectral shape remained unaffected. More specifically, the CPL spectral shape of **EuL** ^**4**^ (Figure 8A) matched **EuL** ^**1**^ within the experimental error (Figure 8G). Similarly, the *g*_lum_ plots (Figure 8F) of the two complexes nearly matched (Figure 8H), whereas **EuL** ^**4**^ produced marginally lower maximal *g*_lum_ values of +0.29 (***Δ*‐EuL** ^**4**^) and −0.28 (***Λ*‐EuL** ^**4**^) (both at 601 nm) for *ΔJ =* 1 and −0.066 (***Δ*‐EuL** ^**4**^ at 607 nm) and +0.069 (***Λ*‐EuL** ^**4**^ at 607.5 nm) for *ΔJ = 2* (**EuL** ^**1**^ produced ±0.290 at 601 nm and ±0.071 at 607.5 nm).

## 4 Conclusion and Future Work

The comparison of the CPL properties across the **EuL** ^**0**^, **EuL** ^**1**^, **EuL** ^**2**^, and **EuL** ^**4**^ series provided a great insight into the relationship between the CPL spectral shape and structural features of the complexes. It was established that only those structural modifications that were proximal to the europium(III) coordination site can significantly impact the luminescent properties. The electronic transitions that give rise to photoemission relax europium(III) from the ^5^ *D*_0_ electronic excited state to different ^7^ *F*_*J*_ states, each containing 2 *J* + 1 degenerate Stark states for each value of *J*. When the 4*f* orbitals of europium(III) experience the CF of the ligand, the degeneracy of the Stark states is lifted, resulting in a characteristic fingerprint spectral shape of each *ΔJ* emission manifold. Therefore, in order to alter the spectral shape, the CF experienced by the 4*f* orbitals must be changed by structural modifications at the europium(III) coordination site or close to it. This was observed when the phenylphosphinate donor groups in **EuL** ^**0**^ were swapped with carboxylate groups in **EuL** ^**1**^, resulting in a substantial change in photophysical properties. The further structural modifications of the top aryl component of the chromophore, such as introduction of a nitro group in **EuL** ^**2**^ and functionalization of the para‐alkoxy group in **EuL** ^**3**^ did not result in any significant changes in the luminescent properties of the europium(III) center. To further investigate this observation, the overall symmetry of the complex was lowered from trigonal to triclinic by modifying one of the three chromophores in **EuL** ^**4**^. This, as expected, did not affect the luminescent properties, including CPL. Such independence of complex luminescence on the extended structure of the chromophore away from the europium(III) coordination site suggested excellent versatility of such materials due to their compatibility with complex‐stage functionalization. In other words, not only **EuL** ^**3**^ and **EuL** ^**4**^ maintain all their photophysical properties in different solvents but also after undergoing complex‐stage functional group interconversion. This suggested a possibility of application in a number of areas such as security inks, bioimaging, optoelectronics, and other applications that would benefit from such bright environmentally independent CPL emitters that can be covalently linked to practically any functional moiety containing one of the common potent functional groups such as amine, thiol, carboxylic acid, or alkyne. Although the nitro group introduced in **EuL** ^**2**^ produced a significant change in such sensitizer‐dependent photophysical properties as *ε*, *Φ*, and emission lifetime, its total emission and CPL spectral shapes, as well as *g*_lum_ values remained the same. Because *Φ* of **EuL** ^**2**^ decreased ~4 times compared to **EuL** ^**1**^, whereas the *τ* remained essentially the same as that of **EuL** ^**1**^, it was suggested that the nitro group, which is conjugated with the rest of the chromophore's *π*‐network facilitated additional NRR pathways, for example, vibrational relaxation via solvent bond oscillators, during the sensitization process prior to the population of the ^5^ *D*_0_ emitting state of europium(III). Since vibrational relaxation is a temperature‐dependent process, **EuL** ^**2**^ could be further studied as a temperature sensor. Because observed emission intensity is proportional to *Φ*, the **EuL** ^**2**^ temperature sensor could exploit it as a variable parameter, whereas the constant *g*_lum_ could serve as an internal reference to facilitate self‐calibrating feature. Other external environmental factors such as pH, solvent polarity, and oxygen concentration that could affect the electronic structure and energy of the chromophore ICT could also be investigated.

## 5 Statistics and Reproducibility

Where instruments incorporating a scanning monochromator have been used (absorption, emission, and excitation spectra) each sample has been recorded and averaged as triplicate measurements. Spectra, where CCD detectors have been employed, have been measured as an average of 100 to a thousand spectra on triplicate samples according to the protocol detailed in the SI section.

## Supporting information


**Table S1:** Time‐dependent solvent gradients for HCPL and UPLC.
**Table S2:** *CPB*_*i*_ calculation for the *ΔJ* = 1 transition of **EuL** ^**0**^ and **EuL** ^**1**^ in MeCN.
**Figure S2:1**: The structure of the chiral stationary phases of *CHIRALPAK IC* and *IE* HPLC columns.
**Figure S2:2**: Chiral HPLC absorbance chromatogram (AC) (at 360 nm) for the chiral separation of **EuL** ^**0**^ enantiomers using *CHIRALPAK IC* (Performed by Dr. Aileen Congreve).
**Figure S2:3:** Chiral HPLC ACs (at 360 nm) for the chiral separation of the **EuL** ^**1**^ enantiomers using *CHIRALPAK IE* (solvent: EtOH/MeOH/TEA/TFA at 50/50/0.5/0.3, injection: 10 μL of *~*0.5 mg ml^−1^ in MeOH, 2 mL min^−1^). Performed by Dr. Aileen Congreve.
**Figure S2:4**: AC (at 340 nm) for the separation of the **EuL** ^**2**^ enantiomers (*CHIRALPAK IE*, solvent: EtOH/MeOH/TEA/TFA at 50/50/0.5/0.3, injection: 20 μL of ~0.5 mg mL^−1^ in MeCN, 2 mL min^−1^) (right). Performed by Dr. Aileen Congreve.
**Figure S2:5**: Chiral HPLC AC (at 360 nm) for the separation of the **EuL** ^**3**^ enantiomers solvent: EtOH/MeOH/TEA/TFA at 50/50/0.5/0.3, injection: 10 μL of ~0.5 mg mL‐1 in MeCN, 2 mL min‐1) (right). Performed by Dr. Aileen Congreve.
**Figure S2:6:** Chiral HPLC ACs (at 360 nm) for the chiral separation of the **EuL** ^**4**^ enantiomers using *CHIRALPAK IE* (solvent: EtOH/MeOH/TEA/TFA at 50/50/0.5/0.3, injection: 10 μL of *~*0.5 mg mL^−1^ in MeOH, 2 mL min^−1^). Performed by Dr. Aileen Congreve.
**Figure S5:1** ^1^H NMR (400 MHz, CDCl_3_) spectrum of ethyl (6‐methyl‐4‐nitropyridin‐2‐yl)(phenyl)phosphinate.
**Figure S5:2**: LCMS (Method E) of ethyl (4‐bromo‐6‐methylpyridin‐2‐yl)(phenyl)phosphinate AC (210–400 nm) (top) and the MS (ESI^+^) at 2.275 min (bottom).
**Figure S5:3** ^1^H NMR (400 MHz, CDCl_3_) spectrum of ethyl (4‐bromo‐6‐methylpyridin‐2‐yl)(phenyl)phosphinate.
**Figure S5:4**: LCMS (Method E) of ethyl (4‐bromo‐6‐methyl‐1‐oxopyridin‐2‐yl)(phenyl)phosphinate AC (210–400 nm) (top) and the MS (ESI^+^) at 1.763 min (bottom).
**Figure S5:5:** ^1^H NMR (400 MHz, CDCl_3_) spectrum of ethyl (4‐bromo‐6‐methyl‐1‐oxopyridin‐2‐yl)(phenyl)phosphinate.
**Figure S5:6:** LCMS (Method E) of ethyl [4‐bromo‐6‐(hydroxymethyl)pyridin‐2‐yl](phenyl) phosphinate AC (210–400 nm) (top) and the MS (ESI^+^) at 1.785 min (bottom).
**Figure S5:7** ^1^H NMR (400 MHz, CDCl_3_) spectrum of ethyl [4‐bromo‐6‐(hydroxymethyl)pyridin‐2‐yl](phenyl) phosphinate.
**Figure S5:8:** LCMS (Method E) of 2‐(2,2‐dibromovinyl)‐1,3,5‐trimethoxybenzene AC (210–400 nm) (top) and the MS (ESI^+^) at 2.875 min (bottom).
**Figure S5:9** ^1^H NMR (400 MHz, CDCl_3_) spectrum of 2‐(2,2‐dibromovinyl)‐1,3,5‐trimethoxybenzene.
**Figure S5:10**: LCMS (Method E) of 2‐ethynyl‐1,3,5‐trimethoxybenzene AC (210–400 nm) (top) and the MS (ESI^+^) at 1.883 min (bottom).
**Figure S5:11** ^1^H NMR (400 MHz, CDCl_3_) spectrum of 2‐ethynyl‐1,3,5‐trimethoxybenzene.
**Figure S5:12**: LCMS (Method E) of ethyl [6‐(hydroxymethyl)‐4‐[2‐(2,4,6‐trimethoxyphenyl)ethynyl] pyridin‐2‐yl](phenyl)phosphinate AC (210–400 nm) (top) and the MS (ESI^+^) at 2.312 min (bottom).
**Figure S5:13** ^1^H NMR (400 MHz, CDCl_3_) spectrum of ethyl [6‐(hydroxymethyl)‐4‐[2‐(2,4,6‐trimethoxyphenyl) ethynyl] pyridin‐2‐yl](phenyl)phosphinate.
**Figure S5:14** ^1^H NMR (400 MHz, CDCl_3_) spectrum of {4‐[2‐(2,4,6‐trimethoxyphenyl)ethynyl]‐6‐[ethoxy(phenyl)phosphoryl]pyridin‐2yl}methyl methanesulfonate.
**Figure S5:15**: LCMS (Method E) of 1,4,7‐tris({4‐[2‐(2,4,6‐trimethoxyphenyl)ethynyl]‐6[ethoxy(phenyl)phosphoryl] pyridin‐2‐yl}methyl)‐1,4,7‐triazacy clononane AC (210–400 nm) (top) and the MS (ESI^+^) at 3.078 min (bottom).
**Figure S5:16**: LCMS (Method E) of **EuL** ^**0**^ AC (210–400 nm) (top) and the MS (ESI^+^) at 2.882 min (bottom).
**Figure S5:17**: Simulated (top) and recorded (bottom) accurate MS (ESI^+^) of **EuL** ^**0**^.
**Figure S5:18**: Accurate MS (ESI^+^) of diethyl 4‐bromopyridine‐2,6‐dicarboxylate.
**Figure S5:19**: LCMS (Method E) of diethyl 4‐bromopyridine‐2,6‐dicarboxylate AC (210–400 nm) (top) and the MS (ESI^+^) at 2.362 min (bottom).
**Figure S5:20** ^1^H NMR (400 MHz, CDCl_3_) spectrum of diethyl 4‐bromopyridine‐2,6‐dicarboxylate.
**Figure S5:21**: LCMS (Method E) of ethyl 4‐bromo‐6‐(hydroxymethyl)picolinate AC (210–400 nm) (top) and the MS (ESI^+^) at 2.345 min (bottom).
**Figure S5:22** ^1^H NMR (400 MHz, CDCl_3_) spectrum of ethyl 4‐bromo‐6‐(hydroxymethyl)picolinate.
**Figure S5:23**: Accurate MS (ESI^+^) of ethyl 6‐(hydroxymethyl)‐4‐((2,4,6‐trimethoxyphenyl)ethynyl)picolinate.
**Figure S5:24**: LCMS (Method E) of ethyl 6‐(hydroxymethyl)‐4‐((2,4,6‐trimethoxyphenyl)ethynyl)picolinate AC (210–400 nm) (top) and the MS (ESI^+^) at 2.282 min (bottom).
**Figure S5:25** ^1^H NMR (600 MHz, CDCl_3_) (top) and ^13^C NMR (151 MHz, CDCl_3_) (bottom) spectra of ethyl 6‐(hydroxymethyl)‐4‐((2,4,6‐trimethoxyphenyl)ethynyl)picolinate.
**Figure S5:26**: LCMS (Method E) of ethyl 6‐(((methylsulfonyl)oxy)methyl)‐4‐((2,4,6‐trimethoxyphenyl) ethynyl)picolinate AC (210–400 nm) (top) and the MS (ESI^+^) at 3.093 min (bottom).
**Figure S5:27** ^1^H NMR (400 MHz, CDCl_3_) spectrum of ethyl 6‐(((methylsulfonyl)oxy)methyl)‐4‐((2,4,6‐trimethoxyphenyl)ethynyl)picolinate.
**Figure S5:28**: Accurate MS (ESI^+^) of triethyl 6,6′,6″‐((1,4,7‐triazonane‐1,4,7‐triyl)tris(methylene))tris(4‐((2,4,6‐trimethoxyphenyl)ethynyl)picolinate).
**Figure S5:29**: Simulated (top) and recorded (bottom) accurate MS (ESI^+^) of **EuL** ^**1**^.
**Figure S5:30**: LCMS (Method E) of **EuL** ^**1**^ AC (210–400 nm) (top) and the MS (ESI^+^) at 2.445 min (bottom).
**Figure S5:31**: Accurate MS (ESI^+^) of 2,4,6‐trimethoxy‐3‐nitrobenzaldehyde.
**Figure S5:32**: LCMS (Method E) of 2,4,6‐trimethoxy‐3‐nitrobenzaldehyde AC (210–400 nm) (top) and the MS (ESI^+^) at 1.992 min (bottom).
**Figure S5:33** ^1^H NMR (700 MHz, CDCl_3_) (top) and ^13^C NMR (151 MHz, CDCl_3_) (bottom) spectra of 2,4,6‐trimethoxy‐3‐nitrobenzaldehyde.
**Figure S5:34**: Accurate MS (ESI^+^) of 2‐(2,2‐dibromovinyl)‐1,3,5‐trimethoxy‐4‐nitrobenzene.
**Figure S5:35**: LCMS (Method E) of 2‐(2,2‐dibromovinyl)‐1,3,5‐trimethoxy‐4‐nitrobenzene AC (210–400 nm) (top) and the MS (ESI^+^) at 2.888 min (bottom).
**Figure S5:36** ^1^H NMR (700 MHz, CDCl_3_) (top) and ^13^C NMR (151 MHz, CDCl_3_) (bottom) spectra of 2‐(2,2‐dibromovinyl)‐1,3,5‐trimethoxy‐4‐nitrobenzene.
**Figure S5:37**: Accurate MS (ESI^+^) of 1‐ethynyl‐2,4,6‐trimethoxy‐3‐nitrobenzene.
**Figure S5:38**: LCMS (Method E) of 1‐ethynyl‐2,4,6‐trimethoxy‐3‐nitrobenzene AC (210–400 nm) (top) and the MS (ESI^+^) at 2.275 min (bottom).
**Figure S5:39** ^1^H NMR (700 MHz, CDCl_3_) (top) and ^13^C NMR (151 MHz, CDCl_3_) (bottom) spectra of 1‐ethynyl‐2,4,6‐trimethoxy‐3‐nitrobenzene.
**Figure S5:40**: Accurate MS (ESI^+^) of ethyl 6‐(hydroxymethyl)‐4‐((2,4,6‐trimethoxy‐3‐nitrophenyl) ethynyl)picolinate.
**Figure S5:41**: LCMS (Method E) of ethyl 6‐(hydroxymethyl)‐4‐((2,4,6‐trimethoxy‐3‐nitrophenyl) ethynyl)picolinate AC (210–400 nm) (top) and the MS (ESI^+^) at 2.382 min (bottom).
**Figure S5:42** ^1^H NMR (700 MHz, CDCl_3_) (top) and ^13^C NMR (151 MHz, CDCl_3_) (bottom) spectra of ethyl 6‐(hydroxymethyl)‐4‐((2,4,6‐trimethoxy‐3‐nitrophenyl) ethynyl)picolinate.
**Figure S5:43**: LCMS (Method E) of ethyl 6‐(((methylsulfonyl)oxy)methyl)‐4‐((2,4,6‐trimethoxy‐3‐nitrophenyl)ethynyl)picolinate AC (210–400 nm) (top) and the MS (ESI^+^) at 2.653 min (bottom).
**Figure S5:44**: Accurate MS (ESI^+^) of triethyl 6,6′,6″‐((1,4,7‐triazonane‐1,4,7‐triyl)tris(methylene))tris(4‐((2,4,6‐trimethoxy‐3‐nitrophenyl)ethynyl)picolinate).
**Figure S5:45**: LCMS (Method E) of **EuL** ^**2**^ AC (210–400 nm) (top) and the MS (ESI^+^) at 2.601 min (bottom).
**Figure S5:46**: Simulated (top) and recorded (bottom) accurate MS (ESI^+^) of **EuL** ^**2**^.
**Figure S5:47** ^1^H NMR (400 MHz, MeOD) (top) and ^13^C NMR (100 MHz, MeOD) (bottom) spectra of 4‐hydroxy‐2,6‐dimethoxybenzaldehyde.
**Figure S5:48**: LCMS (Method E) of 4‐hydroxy‐2,6‐dimethoxybenzaldehyde AC (210–400 nm) (top) and the MS (ESI^+^) at 1.164 min (bottom).
**Figure S5:49**: Accurate MS (ESI^+^) of 4‐(2,2‐dibromovinyl)‐3,5‐dimethoxyphenol.
**Figure S5:50**: LCMS (Method E) of 4‐(2,2‐dibromovinyl)‐3,5‐dimethoxyphenol AC (210–400 nm) (top) and the MS (ESI^+^) at 2.608 min (bottom).
**Figure S5:51** ^1^H NMR (400 MHz, CDCl_3_) (top) and ^13^C NMR (100 MHz, CDCl_3_) (bottom) spectra of 4‐(2,2‐dibromovinyl)‐3,5‐dimethoxyphenol.
**Figure S5:68**: Accurate MS (ESI^+^) of **29**.
**Figure S5:52**: LCMS (Method E) of 4‐ethynyl‐3,5‐dimethoxyphenol AC (210–400 nm) (top) and the MS (ESI^+^) at 1.583 min (bottom).
**Figure S5:53**: Accurate MS (ESI^+^) of 5‐(4‐bromobutoxy)‐2‐ethynyl‐1,3‐dimethoxybenzene.
**Figure S5:54**: LCMS (Method E) of 5‐(4‐bromobutoxy)‐2‐ethynyl‐1,3‐dimethoxybenzene AC (210–400 nm) (top) and the MS (ESI^+^) at 2.858 min (bottom).
**Figure S5:55** ^1^H NMR (400 MHz, CDCl_3_) (top) and ^13^C NMR (100 MHz, CDCl_3_) (bottom) spectra of 5‐(4‐bromobutoxy)‐2‐ethynyl‐1,3‐dimethoxybenzene.
**Figure S5:56**: Accurate MS (ESI^+^) of 5‐(4‐azidobutoxy)‐2‐ethynyl‐1,3‐dimethoxybenzene.
**Figure S5:57**: LCMS (Method E) of 5‐(4‐azidobutoxy)‐2‐ethynyl‐1,3‐dimethoxybenzene AC (210–400 nm) (top) and the MS (ESI^+^) at 2.667 min (bottom).
**Figure S5:58**: Accurate MS (ESI^+^) of ethyl 4‐((4‐(4‐azidobutoxy)‐2,6‐dimethoxyphenyl)ethynyl)‐6‐(hydroxymethyl).
picolinate.
**Figure S5:59**: LCMS (Method E) of ethyl 4‐((4‐(4‐azidobutoxy)‐2,6‐dimethoxyphenyl)ethynyl)‐6‐(hydroxymethyl).
picolinate AC (210–400 nm) (top) and the MS (ESI^+^) at 2.800 min (bottom).
**Figure S5:60** ^1^H NMR (700 MHz, CDCl_3_) (top) and ^13^C NMR (151 MHz, CDCl_3_) (bottom) spectra of ethyl 4‐((4‐(4‐azidobutoxy)‐2,6‐dimethoxyphenyl)ethynyl)‐6‐(hydroxymethyl)picolinate.
**Figure S5:61**: Accurate MS (ESI^+^) of ethyl 4‐((4‐(4‐azidobutoxy)‐2,6‐dimethoxyphenyl)ethynyl)‐6‐(((methylsulfonyl)oxy)methyl)picolinate.
**Figure S5:62**: LCMS (Method E) of ethyl 4‐((4‐(4‐azidobutoxy)‐2,6‐dimethoxyphenyl)ethynyl)‐6‐(((methylsulfonyl)oxy)methyl)picolinate AC (210–400 nm) (top) and the MS (ESI^+^) at 3.108 min (bottom).
**Figure S5:63**: Accurate MS (ESI^+^) of triethyl 6,6′,6″‐((1,4,7‐triazonane‐1,4,7‐triyl)tris(methylene))tris(4‐((4‐(4‐azidobutoxy)‐2,6‐dimethoxyphenyl)ethynyl)picolinate).
**Figure S5:64**: Simulated (top) and recorded (bottom) accurate MS (ESI^+^) of **EuL** ^**3**^.
**Figure S5:65**: LCMS (Method E) of **EuL** ^**3**^ AC (210–400 nm) (top) and the MS (ESI^+^) at 3.258 min (bottom).
**Figure S5:66** ^1^H NMR (400 MHz, CDCl_3_) spectrum of 1‐propargyl‐*β*‐d‐glucopyranoside.
**Figure S5:67**: Simulated (top) and recorded (bottom) accurate MS (ESI^+^) of **EuL** ^**3g**^ ([M + 2H]^2+^, *m*/2).
**Figure S5:68**: LCMS (Method E) of **EuL** ^**3g**^ AC (210–400 nm) (top) and the MS (ESI^+^) at 1.742 min (bottom).
**Figure S5:69**: Accurate MS (ESI^+^) of ethyl 4‐(4‐ethynyl‐3,5‐dimethoxyphenoxy)butanoate.
**Figure S5:70**: LCMS (Method E) of ethyl 4‐(4‐ethynyl‐3,5‐dimethoxyphenoxy)butanoate AC (210–400 nm) (top) and the MS (ESI^+^) at 2.473 min (bottom).
**Figure S5:71** ^1^H NMR (700 MHz, CDCl_3_) (top) and ^13^C NMR (151 MHz, CDCl_3_) (bottom) spectra of ethyl 4‐(4‐ethynyl‐3,5‐dimethoxyphenoxy)butanoate.
**Figure S5:72**: Accurate MS (ESI^+^) of ethyl 4‐((4‐(4‐ethoxy‐4‐oxobutoxy)‐2,6‐dimethoxyphenyl)ethynyl)‐6‐(hydroxymethyl)picolinate.
**Figure S5:73**: LCMS (Method E) of ethyl 4‐((4‐(4‐ethoxy‐4‐oxobutoxy)‐2,6‐dimethoxyphenyl)ethynyl)‐6‐(hydroxymethyl)picolinate AC (210–400 nm).
**Figure S5:74** ^1^H NMR (700 MHz, CDCl_3_) (top) and ^13^C NMR (151 MHz, CDCl_3_) (bottom) spectra of ethyl 4‐((4‐(4‐ethoxy‐4‐oxobutoxy)‐2,6‐dimethoxyphenyl)ethynyl)‐6‐(hydroxymethyl)picolinate.
**Figure S5:75**: Accurate MS (ESI^+^) of **37**.
**Figure S5:76:** LCMS (Method E) of ethyl 4‐((4‐(4‐ethoxy‐4‐oxobutoxy)‐2,6‐dimethoxyphenyl)ethynyl)‐6‐(((methylsulfonyl)oxy)methyl)picolinate AC (210–400 nm) (top) and the MS (ESI^+^) at 2.900 min (bottom).
**Figure S5:77**: LCMS (Method E) of **EuL** ^**4**^ ligand precursor AC (210–400 nm) (top) and the MS (ESI^+^) at 3.158 min (bottom).
**Figure S5:78**: Simulated (top) and recorded (bottom) accurate MS (ESI^+^) of **EuL** ^**4**^.
**Figure S5:79**: LCMS (Method E) of **EuL** ^**4**^ AC (210–400 nm) (top) and the MS (ESI^+^) at 2.900 min (bottom).

## Data Availability

All data collected and analyzed during this study including spectra and drawings are available from the corresponding author upon request.
