# Supplementary material for: Investigating the CPL‐Structure Dependence in Arylalkynylpyridyl—Triazacyclononane Sensitized Nine‐Coordinate Europium(III) Complexes
Source: Chirality. 2026 Sep 7;38(9):e70145. doi: 10.1002/chir.70145 (PMC13549766; doi:10.1002/chir.70145)
Supplement: Supplementary file 1 [file CHIR-38-e70145-s001.docx]

**Investigating the CPL-structure dependence in arylalkynylpyridyl - triazacyclononane sensitised nine-coordinate europium(III) complexes**

***Artemijs Krimovs^1^, Robert Pal^1^****

^1^Department of Chemistry, Durham University, South Road, Durham, DH1 3LE, UK

*robert.pal@durham.ac.uk

Supplementary Information

**Table of Contents**

**1. General procedures**	2

**2. HPLC separation**	2

2.1. Preparative and analytical reverse-phase HPLC and UPLC	2

2.2. Chiral HPLC.	4

**3. Photophysical measurements**	6

3.1. Absorption, emission and lifetime measurements	6

3.2. CPL spectroscopy	6

3.3. Circularly polarised brightness calculation	7

**4. Supplementary Schemes**	8

**5. Synthetic Procedures**	11

**Supplementary References**	77

1. General procedures

All starting materials are commercially available and distributed by *Merck*. Analytical grade and HPLC grade solvents were used and degassed using freeze-pump-thaw or cycling where appropriate.

Silica gel plates on aluminium support (0.2 mm thick, 60 F254, *Merck*) were used in thin layer chromatography (TLC) and resolved under UV radiation source at 254 or 365 nm. Flash column chromatography was manually prepared using silica gel (60 (230-400 mesh, *Flourochem*).

^1^H and ^13^C NMR was performed on a *Bruker Advance-400* (^1^H at 400.06 MHz and^13^C at 100.61 MHz), a *Mercury 400* (^1^H at 399.95 MHz), a *Varian VNMRS-600* (^1^H at 599.67 MHz and ^13^C at 150.79 MHz) or a *Varian VNMRS-700* (^1^H at 699.73 MHz and ^13^C at 175.95 MHz) using CDCl_3_ and MeOD (*Merck*). Chemical shifts and J-couplings were reported in ppm and Hz respectively.

LCMS experiments were performed on Waters Single Quadrupole Detector (SQD) spectrometer exploiting *Acquity* ultra-performance liquid chromatography (UPLC) and electrospray ionisation (ESI) source. The instrument is set up for alternate positive / negative ion MS scans plus PD array detection. The UPLC exploits *Acquity* UPLC ethylene bridged hybrid C_18_ 1.7 μm (2.1 × 50 mm) analytical column with MeCN – water gradient (Method E – section VI.2, Table 6.1). Accurate mass measurements were performed on Waters Quadrupole Time-of-Flight (QToF) mass spectrometer configured for ESI and coupled to an *Acquity* UPLC.

2. HPLC separation

2.1. Preparative and analytical reverse-phase HPLC and UPLC

Preparative reverse-phase HPLC was performed using a Shimadzu module comprising of a vacuum degasser (*DGU-20AR5*), a *Prominence* liquid chromatography pump *(LC-20AP*), a *Prominence* UV-Vis detector (*SPD-20A*) and a communications bus module (*CBM-20A*). Sample separation was performed using a preparative *XBridge* C_18_ OBD column (19 × 100 mm, 5 μm, flow rate = 17 cm^3^ min^-1^) with fractions collected manually. The solvent system was a mixture of water and MeCN, where the proportion of the two solvents was varied in time (Table 6.1, Methods A-D). Analytical UPLC was performed as part of LCMS experiments as per above (Section VI.1.).

**Table S1**: Time dependent solvent gradients for HCPL and UPLC.

| Method | Time/ mins | Water fraction /% | MeCN fraction /% |
| --- | --- | --- | --- |
| **Method A**  (10 min gradient from 10%) | 0 | 90 | 10 |
|  | 4 | 90 | 10 |
|  | 14 | 0 | 100 |
|  | 19 | 0 | 100 |
|  | 21 | 90 | 10 |
| **Method B** (15 min gradient from 10%) | 0 | 90 | 10 |
|  | 4 | 90 | 10 |
|  | 19 | 0 | 100 |
|  | 24 | 0 | 100 |
|  | 27 | 90 | 10 |
| **Method C** (20 min gradient from 10%)* | 0 | 90 | 10 |
|  | 4 | 90 | 10 |
|  | 24 | 0 | 100 |
|  | 29 | 0 | 100 |
|  | 32 | 90 | 10 |
| **Method D** (15 min gradient from 30%) | 0 | 70 | 30 |
|  | 4 | 70 | 30 |
|  | 19 | 0 | 100 |
|  | 23 | 0 | 100 |
|  | 26 | 70 | 30 |
| **Method E** (Analytical)* | 0 | 95 | 5 |
|  | 0.2 | 95 | 5 |
|  | 4 | 5 | 95 |
|  | 4.5 | 5 | 95 |
|  | 5 | 95 | 5 |

*0.1% formic acid in water was used instead of water

2.2. Chiral HPLC.

Chiral HPLC was performed using a *Perkin Elmer Series 200* module consisting of a *Perkin Elmer Series 200* pump, autosampler, UV-Vis detector and *CHIRALPAK* *IC* column (10 × 250 mm, flow rate = 4.4 ml min^-1^, isocratic MeOH) for **EuL^0^** and either *CHIRALPAK* IE columns (4.6 × 250 mm, flow rate = 2 ml min^-1^ at 40 °C, EtOH/MeOH/TEA/TFA at 50/50/0.5/0.3 ratio) for the rest of the materials.

**Figure S2.1**: The structure of the chiral stationary phases of *CHIRALPAK IC* and *IE* HPLC columns.

**Figure S2.2**: Chiral HPLC absorbance chromatogram (AC) (at 360 nm) for the chiral separation of separation of **EuL^0^** enantiomers using *CHIRALPAK IC* (Performed by Dr. Aileen Congreve.

**Figure S2.3:** Chiral HPLC ACs (at 360 nm) for the chiral separation of the **EuL^1^** enantiomers using *CHIRALPAK IE* (solvent: EtOH/MeOH/TEA/TFA at 50/50/0.5/0.3, injection: 10 μL of *~*0.5 mg ml^-1^ in MeOH, 2 ml min^-1^). Performed by Dr. Aileen Congreve.

**Figure S2.4**: AC (at 340 nm) for the separation of the **EuL^2^** enantiomers (*CHIRALPAK* *IE*, solvent: EtOH/MeOH/TEA/TFA at 50/50/0.5/0.3, injection: 20 μL of ~0.5 mg ml^-1^ in MeCN, 2 ml min^-1^) (right). Performed by Dr. Aileen Congreve.

**Figure S2.5**: Chiral HPLC AC (at 360 nm) for the separation of the **EuL^3^** enantiomers solvent: EtOH/MeOH/TEA/TFA at 50/50/0.5/0.3, injection: 10 μL of ~0.5 mg ml-1 in MeCN, 2 ml min-1) (right). Performed by Dr. Aileen Congreve.

**Figure S2.6:** Chiral HPLC ACs (at 360 nm) for the chiral separation of the **EuL^4^** enantiomers using *using CHIRALPAK IE* (solvent: EtOH/MeOH/TEA/TFA at 50/50/0.5/0.3, injection: 10 μL of *~*0.5 mg ml^-1^ in MeOH, 2 ml min^-1^). Performed by Dr. Aileen Congreve.

3. Photophysical measurements

3.1. Absorption, emission and lifetime measurements

All solution state optical analyses were carried out in quartz cuvettes with a path length of 1.0000 cm. Solid state (PMMA) samples were recorded using a custom-built holder. UV-vis absorption was measured on an *ATI Unicam* UV-vis spectrometer using *Vision* software (version 3.33). Emission experiments were performed on an *ISA Jobin-Yvon Spex* *Fluorolog-3* luminescence spectrometer using *DataMax* software (version 2.2.10). Lifetime was measured with a *Perkin Elmer LS55* spectrometer using *FL Winlab* software. All emission experiments were performed at 1 μM solution concentration. Absolute photoluminescent quantum yields were determined using a *Horiba FluoroMax Plus* fitted with a *QuantaPhi-2* integrating sphere. Excitation and emission slits were fixed at 2 nm with neutral density filters (*ThorLabs, NEK01S*) used at the excitation aperture to maintain a signal of approximately 10^6^ cps at the excitation wavelength.

3.2. CPL spectroscopy

CPL was measured with a home-built (modular) spectrometer.^1^ The excitation source was a broad band (200 – 1000 nm) nitrogen purged, laser ­- driven light source *EQ 99* (*Elliot Scientific*). The excitation wavelength was selected by feeding the broadband light into an *Acton SP-2155* monochromator (*Princeton Instruments*); the collimated light was focused into the sample cell (1.0000 cm quartz cuvette). Sample PL emission was collected perpendicular to the excitation direction with a lens (f = 150 mm). The emission was fed through a PEM (*Hinds Series II/FS42AA*) and through a linear sheet polariser (*Comar*). The light was then focused into a second SM (*Acton SP2155*) and subsequently on to a PMT (*Hamamatsu H10723 series*). The detection of the CPL signal was achieved using the field modulation lock-in technique. The electronic signal from the PMT was then fed into a lock-in amplifier (*Hinds Instruments Signaloc Model* 2100). The reference signal for the lock-in detection was provided by the PEM control unit. The monochromators, PEM control unit and lock-in amplifier were interfaced to a desktop PC and controlled by a custom-written *LabVIEW* *2014b* graphic user interface. The lock-in amplifier provided two signals, an alternating current signal corresponding to (*I*_L_- *I*_R_) and a direct current signal corresponding to (*I*_L_+ *I*_R_) after background subtraction. Spectral calibration of the scanning monochromator was performed using a
Hg-Ar calibration lamp (*Ocean Optics*, *HG-2*). A correction factor for the wavelength dependence of the detection system was constructed using a calibrated lamp (*Ocean Optics*, *HL-2000*). The measured raw data was subsequently corrected using this correction factor. The validation of the CPL detection systems was achieved using LEDs at various emission wavelengths (i.e. 650/50 nm). The LED was mounted in the sample holder and the light from the LED was fed through a broad band polarising filter and QWP (*ThorLabs, LPVISE100-A* and *AQWP05M-600*) to generate CP light. The emission spectra were recorded with 0.5 nm step size, and the slits of the excitation and emission monochromators were set to 1 nm FWHM. CPL and total emission spectra were obtained through an averaging procedure of 5 scans. Prior to all measurements, the QWP and an LED were used to set the phase of the lock-in amplifier correctly.

3.3. Circularly polarised brightness calculation

The circularly polarised brightness (*CPB)* combines the dissymmetry factor (*g*_lum_) and brightness (*B*, the product of *ε* and *φ*) of a compound:^2^

$$\begin{aligned} CPB= \varepsilon\Phi\frac{g_{\text{lum}}}{2} \#\left[ Equation 1 \right] \end{aligned}$$

High values indicate the compound can emit large quantities of one handed circularly polarised photons. Therefore, *CPB* can be used as a reliable reference parameter to compare the performance of different CPL-active materials. *CPB* can also be calculated for individual transition (*CPB_i_*) using branching factor (*β*_ι_) which is the ratio between the intensities of the emission band of interest (*I_i_*) and total emission:

$$\begin{aligned} {CPB}_{i}=\beta_{i}\times CPB \#\left[ Equation 2 \right] \end{aligned}$$

It is particularly useful for applications where the wavelength of the detected light can be selected, for instance, by a band pass filter.^3^ In that case, the average *g*_lum_ for the selected wavelength region is used to calculate *CPB_i_.* Therefore, the emission manifold with single CPL sign is desired to maximise *CPB_i_* by avoiding cancellation of CPL signal.

**Table S2**: *CPB_i_* calculation for the *ΔJ* = 1 transition of **EuL^0^** and **EuL^1^** in MeCN.

| Compound | Trans.  *λ* range /nm | *β_i_* /% | max  \|*g*_lum_\| | *B  /*M^-1^ cm^-1^ | *CPB_i_* /M^-1^ cm^-1^ |
| --- | --- | --- | --- | --- | --- |
| **EuL^0^** | 589-601 | 7.3 | 0.21 | 21600  (at 355 nm) | **166** |
| **EuL^1^** |  | 5.9 | 0.30 | 34650  (at 358 nm) | **307** |

4. Supplementary Schemes

**Scheme S1**: The reaction sequence for the ‘bottom’ of the phenylphospinate donor chromophore for **EuL^0^**.

**Scheme S2:** The reaction sequence for the ‘top’ of the chromophore.

**Scheme S3**: The reaction sequence for the Sonogashira cross-coupling, mesylation, alkylation, hydrolysis and complexation of **EuL^0^**.

**Scheme S4**: The reaction sequence for the ‘bottom’ of the carboxylate donor sensitising chromophore.

**Scheme S5**: The reaction sequence for the carboxylate donor sensitising chromophore for **EuL^1^** and **EuL^4^**.

**Scheme S6**: The reaction sequence for the alkylation, hydrolysis and complexation steps for **EuL^1^**.

**Scheme S7**: The reaction sequence for the sensitising chromophore of **EuL^2^**.

**Scheme S8**: The reaction sequence for the alkylation, hydrolysis and complexation steps for **EuL^2^**.

**Scheme S9:** The reaction sequence for the sensitising chromophore of **EuL^3^.**

**Scheme S10:** The reaction sequence for the alkylation, hydrolysis and complexation steps for **EuL^3^**.

**Scheme S11:** The reaction sequence for the sensitising chromophore of **EuL^4^**.

5. Synthetic Procedures

Ethyl (6-methyl-4-nitropyridin-2-yl)(phenyl)phosphinate^4^

2-bromo-6-methyl-4-nitropyridine (4.00 g, 18.4 mmol, 1 eq.) was dissolved in anhydrous toluene. The solution was degassed (freeze-pump-thaw × 3) before TEA (9 ml), ethyl phenylphosphinate (3.20 ml, 21.2 mmol, 1.2 eq.) and [1,1′-bis(diphenylphosphino) ferrocene]dichloropalladium (II) (0.756 g, 0.926 mmol, 0.05 eq.) were added. The resulting red solution was stirred at 120 °C for 16 hours under argon to produce a black residue. The solvent was then removed under reduced pressure, and the residue dissolved in DCM. The solution was washed with 1 M HCl (20 ml × 3) and water (20 ml × 2), followed by extraction of the combine aqueous layers with DCM (10 ml × 3). The combined organic layers were dried over Na_2_SO_4_ and filtered. The solvent was removed under reduced pressure to result in a black solid that was then purified by flash chromatography (solid loading, silica, 1:3 to 3:2 EtOAc to hexane) to yield a yellow oil (1.15 g, 3.748 mmol, 20%).

^1^H NMR (400 MHz, CDCl_3_) *δ* 8.47 (1 H, dd, ^3^J_H-P_ 6.2, ^4^J_H-H_ 2.0, H^2^), 7.89 (2 H, ddd, ^3^J_H-P_ 12.3, ^3^J_H-H_ 8.2, ^4^J_H-H_ 1.5, H^8^), 7.84 (1 H, t, ^4^J_H-H_ 1.5, H^4^), 7.46 (1 H, td, ^3^J_H-H_ 7.5, ^4^J_H-H_ 1.5, H^10^), 7.38 (2 H, td, ^3^J_H-H_ 7.7, ^4^J_H-P_ 3.8, H^9^), 4.14-4.03 (2 H, m, H^11^), 2.64 (3 H, s, H^6^), 1.29 ( 3 H, t, 7.1, H^12^).

**Figure S5.1**: ^1^H NMR (400 MHz, CDCl_3_) spectrum of ethyl (6-methyl-4-nitropyridin-2-yl)(phenyl)phosphinate.

Ethyl (4-bromo-6-methylpyridin-2-yl)(phenyl)phosphinate^4^

Ethyl(6-methyl-4-nitropyridin-2-yl)(phenyl)phosphinate (1.15 g, 3.748 mmol, 1 eq.) was dissolved in acetyl bromide (12.0 ml, 162 mmol, 43 eq.), and the solution was stirred at 70 °C for 10 hours. After the reaction mixture was allowed to cool to rt., it was dropwise added to stirring ice-cold MeOH (30 ml). Once added, the mixture was stirred at rt for further an additional 1 hour before the solvent was removed under reduced pressure to yield dark red oil. The oil was then dissolved in triethyl orthoformate (30.0 ml, 180 mmol, 48 eq.) and refluxed at 120 °C for 65 hours under argon. The solvent was then removed under reduced pressure to yield brown oil which was purified by flash chromatography (silica, DCM to 0.8 % MeOH in DCM) to yield pale yellow oil (442 mg, 1.30 mmol, 35 %).

LCMS (ESI^+^) *m/z* 340.144 [M+H]^+^.

^1^H NMR (400 MHz, CDCl_3_) *δ* 8.04 (1 H, dd, ^3^J_H-P_ 6.2, ^4^J_H-H_ 1.8, H^2^), 7.95 (2 H, ddd, ^3^J_H-P_ 12.2, ^3^J_H-H_ 8.1, ^4^J_H-H_ 1.5, H^8^), 7.50 (1 H, td, ^3^J_H-H_ 7.5, ^4^J_H-H_ 1.5, H^10^), 7.42 (2 H, td, ^3^J_H-H_ 7.4, ^4^J_H-P_ 3.7, H^9^), 7.36 (1 H, t, ^4^J_H-H_ 1.4, H^4^) 4.18-4.03 (2 H, m, H^11^), 2.51 (3 H, s, H^6^), 1.33 ( 3 H, t,^3^J_H-H_ 7.1, H^12^).

**Figure S5.2**: LCMS (Method E) of ethyl (4-bromo-6-methylpyridin-2-yl)(phenyl)phosphinate AC (210-400 nm) (top) and the MS (ESI^+^) at 2.275 min (bottom).

**Figure S5.3**: ^1^H NMR (400 MHz, CDCl_3_) spectrum of ethyl (4-bromo-6-methylpyridin-2-yl)(phenyl)phosphinate.

Ethyl (4-bromo-6-methyl-1-oxo-pyridin-2-yl)(phenyl)phosphinate^4^

Ethyl (4-bromo-6-methylpyridin-2-yl)(phenyl)phosphinate (442 mg, 1.30 mmol, 1 eq.) and meta-chloroperoxybenzoic acid (500 mg, 2.90 mmol, 2.2 eq.) were dissolved in anhydrous CDCl_3_ (8 ml) and refluxed at 65°C for 20 hours under argon. After that, solvent was removed under reduced pressure to give yellow residue which was redissolved in DCM (15 ml) and washed with 0.5 M sodium bicarbonate (15 ml × 4), followed by extraction of the combined aqueous layers with DCM (15 ml × 3). The combined organic layers were then dried over Na_2_SO_4_, filtered and concentrated. The crude product was purified by flash chromatography (DCM to 2.5 % MeOH in DCM) to yield yellow oil (245 mg, 0.689 mmol, 53%);

LCMS (ESI^+^) *m/z* 356.144 [M+H]^+^.

^1^H NMR (400 MHz, CDCl_3_) *δ* 8.05 (1 H, dd, ^3^J_H-P_ 7.8, ^4^J_H-H_ 2.9, H^2^), 7.97 (2 H, ddd, ^3^J_H-P_ 13.7, ^3^J_H-H_ 8.3, ^4^J_H-H_ 1.5, H^8^), 7.50 (1 H, td, ^3^J_H-H_ 7.5, ^4^J_H-H_ 1.5, H^10^), 7.45 (1 H, d, ^4^J_H-H_ 3.0, H^4^), 7.41 (2 H, td, ^3^J_H-H_ 7.7, ^4^J_H-P_ 4.1, H^9^), 4.17-4.08 (2 H, m, H^11^), 2.32 (3 H, s, H^6^), 1.34 ( 3 H, t, ^3^J_H-H_ 7.1, H^12^).

**Figure S5.4**: LCMS (Method E) of ethyl (4-bromo-6-methyl-1-oxo-pyridin-2-yl)(phenyl)phosphinate AC (210-400 nm) (top) and the MS (ESI^+^) at 1.763 minutes (bottom).

**Figure S5.5:** ^1^H NMR (400 MHz, CDCl_3_) spectrum of ethyl (4-bromo-6-methyl-1-oxo-pyridin-2-yl)(phenyl)phosphinate.

Ethyl [4-bromo-6-(hydroxymethyl)pyridin-2-yl](phenyl) phosphinate^4^

Trifluoroacetic acid anhydride (2.00 ml, 14.4 mmol, 21 eq.) was added to the solution of ethyl (4-bromo-6-methyl-1-oxo-pyridin-2-yl)(phenyl)phosphinate (245 mg, 0.689 mmol, 1 eq.) in anhydrous MeCN (5 ml). The resulting green solution was refluxed at 60°C for 6 h under argon until the formation of trifluoroacetate ester was complete (^1^H NMR: *δ* H^3^ 2.32 → 5.46 ppm). The solvent was removed under reduced pressure, and the residue was redissolved in a 1:1 solution of ethanol and water (10 ml). The resulting solution was stirred at rt for 17 hours followed by evaporation of the organic solvent under reduced pressure. The resulting aqueous solution was extracted with DCM (20 ml × 3), and the combined organic layers were dried over Na_2_SO_4_, filtered and concentrated. The crude product was purified by flash chromatography (silica, DCM to 2.5 % MeOH in DCM) to yield yellow oil (104 mg, 0.292 mmol, 42 %).

^1^H NMR (400 MHz, CDCl_3_) *δ* 8.10 (1 H, dd, ^3^J_H-P_ 6.0, ^4^J_H-H_ 1.8, H^2^), 7.92 (2 H, ddd, ^3^J_H-P_ 12.3, ^3^J_H-H_ 8.3, ^4^J_H-H_ 1.4, H^8^), 7.61 (1 H, t, ^4^J_H-H_ 1.4, H^4^), 7.54 (1 H, td, ^3^J_H-H_ 7.5, ^4^J_H-H_ 1.5, H^10^), 7.46 (2 H, td, ^3^J_H-H_ 7.7, ^4^J_H-P_ 3.7, H^9^), 4.74 (2 H, s, H^6^), 4.19-4.05 (2 H, m, H^11^), 1.36 ( 3 H, t, ^3^J_H-H_ 7.0, H^12^).

LCMS (ESI^+^) *m/z* 356.144 [M+H]^+^.

**Figure S5.6:** LCMS (Method E) of ethyl [4-bromo-6-(hydroxymethyl)pyridin-2-yl](phenyl) phosphinate AC (210-400 nm) (top) and the MS (ESI^+^) at 1.785 minutes (bottom).

**Figure S5.7**: ^1^H NMR (400 MHz, CDCl_3_) spectrum of ethyl [4-bromo-6-(hydroxymethyl)pyridin-2-yl](phenyl) phosphinate.

2-(2,2-Dibromovinyl)-1,3,5-trimethoxybenzene^4^

A solution of CBr_4_ (1.69 g, 5.10 mmol, 2 eq.) in anhydrous DCM (3 ml) was dropwise added to a cooled (ice bath) solution of 2,4,6-trimethoxybenzaldehyde (0.5 g, 2.5 mmol, 1 eq.) and PPh_3_ (5.36 g, 20.5 mmol, 10 eq.) in anhydrous DCM (3 ml), and the resulting solution was stirred at rt for 20 hours under argon. After that, the solution was washed with water
(10 ml × 5) followed by extraction of combined aqueous layers with DCM (10 ml × 3). The combined organic layers were dried over Na_2_SO_4_, filtered and concentrated. The crude product was purified by column chromatography (silica, 30 to 50 % DCM in hexane) to yield white solid (321 mg, 0.912 mmol, 36%).

LCMS (ESI^+^) *m/z* 351.051 [M+H]^+^.

^1^H NMR (400 MHz, CDCl_3_) *δ* 7.19 (1 H, s, H^14^), 6.11 (2 H, s, H^17^), 3.83 (3 H, s, H^20^), 3.81 (6 H, s, H^19^);

**Figure S5.8:** LCMS (Method E) of 2-(2,2-dibromovinyl)-1,3,5-trimethoxybenzene AC (210-400 nm) (top) and the MS (ESI^+^) at 2.875 minutes (bottom).

**Figure S5.9**: ^1^H NMR (400 MHz, CDCl_3_) spectrum of 2-(2,2-dibromovinyl)-1,3,5-trimethoxybenzene**.**

2-Ethynyl-1,3,5-trimethoxybenzene^4^

2-(2,2-dibromovinyl)-1,3,5-trimethoxybenzene (321 mg, 0.912 mmol, 1 eq.) was dissolved in anhydrous THF and cooled to -78 °C (acetone, dry ice). 1.6 M solution of n-butyllithium in hexane (1.5 ml, 2.4 mmol, 2.6 eq.) was then added dropwise, and the resulting orange solution was stirred for 20 minutes at -78 °C. After that, water (4 ml) was added dropwise, and the resulting green solution was stirred at rt for additional 30 minutes. After that, the organic solvents were removed under reduced pressure, and the resulting aqueous solution was extracted with ethyl acetate (20 ml × 3). The combined organic layers were washed with brine, dried over Na_2_SO_4_, filtered and dried under reduced pressure to yield yellow solid that was used without further purification (0.171 g, 0.890 mmol, 98%).

LCMS (ESI^+^) *m/z* 193.104 [M+H]^+^.

^1^H NMR (400 MHz, CDCl_3_) *δ* 6.10 (2 H, s, H^17^), 3.88 (6 H, s, H^19^), 3.83 (3 H, s, H^20^), 3.50 (1 H, s, H^13^).

**Figure S5.10**: LCMS (Method E) of 2-ethynyl-1,3,5-trimethoxybenzene AC (210-400 nm) (top) and the MS (ESI^+^) at 1.883 minutes (bottom).

**Figure S5.11**: ^1^H NMR (400 MHz, CDCl_3_) spectrum of 2-ethynyl-1,3,5-trimethoxybenzene.

Ethyl [6-(hydroxymethyl)-4-[2-(2,4,6-trimethoxyphenyl)ethynyl] pyridin-2-yl](phenyl)phosphinate

2-ethynyl-1,3,5-trimethoxybenzene (0.0613 g, 0.319 mmol 1.1 eq.), ethyl [4-bromo-6-(hydroxymethyl)pyridin-2-yl](phenyl) phosphinate (0.104 g, 0.290 mmol, 1 eq.) and [Pd(allyl)Cl]_2_ (0.0106 g, 0.0290 mmol, 0.1 eq.) were dissolved in anhydrous MeCN (3 ml) and degassed (freeze-pump-thaw × 3). After that, P(^t^Bu)_3_ (20 μl, 0.087 mmol, 0.3 eq.) and piperidine (110 μl,1.16 mmol, 4 eq.) were added, and the resulting solution was stirred at 40°C for 20 hours under argon. Organic solvents were then evaporated under reduced pressure, and the residue was dissolved in DCM (3 ml). The resulting solution was washed with water (5 ml × 3), followed by extraction of the combined aqueous layers with DCM (5 ml × 3). The combined organic layers were then dried over Na_2_SO_4_, filtered and isolated from solvent under reduced pressure. The resulting dark red oil was purified using reverse-phase HPLC (C_18_, Method B, retention time = 10 minutes) to yield yellow oil (32 mg, 0.068 mmol, 23%).

LCMS (ESI^+^) *m/z* 468.292 [M+H]^+^.

^1^H NMR (400 MHz, CDCl_3_) *δ* 8.15 (1 H, d, ^3^J_H-P_ 6.5, H^2^), 7.97 (2 H, ddd, ^3^J_H-P_ 12.3, ^3^J_H-H_ 8.3, ^4^J_H-H_ 1.4, H^8^), 7.54 (1 H, td, ^3^J_H-H_ 7.5, ^4^J_H-H_ 1.5, H^3^), 7.47 (2 H, td, ^3^J_H-H_ 7.7, ^4^J_H-P_ 3.7, H^9^), 7.40 (1 H, t, ^4^J_H-H_ 1.4 H^4^), 6.10 (2 H, s, H^17^), 4.74 (2 H, d, ^4^J_H-H_ 1.4, H^6^), 4.19-4.05 (2 H, m, H^11^), 3.89 (6 H, s, H^19^), 3.85 (3 H, s, H^20^), 1.38 ( 3 H, t, ^3^J_H-H_ 7.1, H^12^);

**Figure S5.12**: LCMS (Method E) of ethyl [6-(hydroxymethyl)-4-[2-(2,4,6-trimethoxyphenyl)ethynyl] pyridin-2-yl](phenyl)phosphinate AC (210-400 nm) (top) and the MS (ESI^+^) at 2.312 minutes (bottom).

**Figure S5.13**: ^1^H NMR (400 MHz, CDCl_3_) spectrum of ethyl [6-(hydroxymethyl)-4-[2-(2,4,6-trimethoxyphenyl) ethynyl] pyridin-2-yl](phenyl)phosphinate.

{4-[2-(2,4,6-Trimethoxyphenyl)ethynyl]-6-[ethoxy(phenyl)phosphoryl]
pyridin-2yl}methyl methanesulfonate^4^

Ethyl [6- (hydroxymethyl)-4-[2-(2,4,6-trimethoxyphenyl) ethynyl] pyridin-2-yl] (phenyl) phosphinate (32 mg, 0.068 mmol, 1 eq.) was dissolved in anhydrous THF (3 ml). *N,N*-Diisopropylethylamine (DIPEA) (120 μl, 0.520 mmol, 7.6 eq.) and methanesulfonic anhydride (48 mg, 0.278 mmol, 4 eq.) were added, and the resulting solution was stirred at rt for 4h under argon. After that, organic solvents were then evaporated under reduced pressure, and the residue dissolved in DCM (10 ml). The resulting solution was washed with water (15 ml × 3), followed by extraction of the combined aqueous layers with DCM (15 ml × 3). The combined organic layers were then dried over Na_2_SO_4_, filtered and isolated from solvent under reduced pressure to yield dark yellow oil that was used in the next step without further purification.

^1^H NMR (400 MHz, CDCl_3_) *δ* 8.22 (1 H, dd, ^3^J_H-P_ 6.2, ^4^J_H-H_ 1.4, H^2^), 7.97 (2 H, ddd, ^3^J_H-P_ 12.3, ^3^J_H-H_ 8.3, ^4^J_H-H_ 1.4, H^8^), 7.60 (1 H, t, ^4^J_H-H_ 1.5, H^4^), 7.57 (1 H, td, ^3^J_H-H_ 7.5, ^4^J_H-H_ 1.5, H^10^), 7.47 (2 H, td, ^3^J_H-H_ 7.7, ^4^J_H-P_ 3.7, H^9^), 6.14 (2 H, s, H^17^), 5.32 (2 H, s, H^6^), 4.19-4.05 (2 H, m, H^11^), 3.93 (6 H, s, H^19^), 3.88 (3 H, s, H^20^), 1.40 ( 3 H, t, ^3^J_H-H_ 7.1, H^12^).

**Figure S5.14**: ^1^H NMR (400 MHz, CDCl_3_) spectrum of {4-[2-(2,4,6-trimethoxyphenyl)ethynyl]-6-[ethoxy(phenyl)phosphoryl]pyridin-2yl}methyl methanesulfonate.

1,4,7-Tris({4-[2-(2,4,6-trimethoxyphenyl)ethynyl]-6[ethoxy(phenyl)phosphoryl]pyridin-2-yl}methyl)-1,4,7-triazacyclononane^4^

Ethyl [6-(hydroxymethyl)-4-[2-(2,4,6-trimethoxyphenyl)ethynyl] pyridin-2yl](phenyl) phosphinate (*0.068* mmol, 3.4 eq.), TACN.3HCl (4.7 mg, 0.020 mmol, 1 eq.) and K_2_CO_3_ (19.2 mg, 0.139 mmol, 7 eq.) were dissolved in anhydrous MeCN (2 ml) and stirred at 60°C for 20 hours under argon. After that, MeCN was removed under reduced pressure, and the crude was redissolved in MeOH for the use in the next step without purification.
LCMS (ESI^+^) *m/z* 1477.840 [M+H]^+^.

**Figure S5.15**: LCMS (Method E) of 1,4,7-tris({4-[2-(2,4,6-trimethoxyphenyl)ethynyl]-6[ethoxy(phenyl)phosphoryl] pyridin-2-yl}methyl)-1,4,7-triazacyclononane AC (210-400 nm) (top) and the MS (ESI^+^) at 3.078 minutes (bottom).

**EuL^0^** ^4^

1,4,7-Tris({4-[2-(2,4,6-trimethoxyphenyl)ethynyl]-6[ethoxy(phenyl)phosphoryl]pyridin-2-yl}methyl)-1,4,7-triazacyclononane (*0.020* mmol) was dissolved in MeOH (2 ml) and water until white precipitate persisted. The pH of the resulting solution was adjusted to 12.5 using 1M NaOH, and the solution was stirred at 60°C for 72 hours, while its pH was monitored and periodically adjusted to 12.5 until hydrolysis completion was confirmed by LCMS. After that, the solution pH was readjusted to 6.5 using 0.5 M HCl. EuCl_3_.6H_2_O was then added (8.0 mg, 0.022 mmol, 1.1 eq.) and the resulting solution was stirred at rt for 18 hours. The solvents were then evaporated under reduced pressure to yield white solid which was purified using reverse-phase HPLC (C18, Method A, retention time = 13.5 minutes) to yield white solid (13 mg, 0.0084 mmol, 42% over three steps).

HRMS (ESI^+^) *m/z* 1541.3539 [M+H]^+^ (C_75_H_72_^151^EuN_6_O_15_P_3_ requires 1541.3545).

**Figure S5.16**: LCMS (Method E) of **EuL^0^** AC (210-400 nm) (top) and the MS (ESI^+^) at 2.882 minutes (bottom).

**Figure S5.17**: Simulated (top) and recorded (bottom) accurate MS (ESI^+^) of **EuL^0^**.

Diethyl 4-bromopyridine-2,6-dicarboxylate^5^

A solid mixture of chelidamic acid (1.64 g, 8.97 mmol, 1 eq.) and phosphorus pentabromide (11.6 g, 26.9 mmol, 3 eq.) was melted and heated at 95 °C for 3 hours. After that, anhydrous CHCl_3_ (30 ml) was added, and the resulting hot mixture was filtered and cooled in an ice bath. This was followed by drop-wise addition of cold EtOH (20 ml) to the reaction mixture which was then stirred at rt for 15 minutes. After that, the solvents were evaporated under reduced pressure to yield brown oil. The oil was then dissolved in ice-cold water and stirred at 0 °C for 1 hour to allow for precipitation of the crude product. The resulting brown solid was dried and recrystallised from hexane to yield light-brown solid (1.23 g, 4.07 mmol, 45%).

HRMS (ESI^+^) *m/z* 302.0023 (C_11_H_13_NO_4_Br requires 302.0028).

^1^H NMR (400 MHz, CDCl_3_) *δ* 8.42 (2 H, s, H^2^), 4.50 (4 H, q, ^3^J_H-H_ 7.1, H^5^), 1.46 (6 H, t, ^3^J_H-H_ 7.4, H^6^).

**Figure S5.18**: Accurate MS (ESI^+^) of diethyl 4-bromopyridine-2,6-dicarboxylate.

**Figure S5.19**: LCMS (Method E) of diethyl 4-bromopyridine-2,6-dicarboxylate AC (210-400 nm) (top) and the MS (ESI^+^) at 2.362 minutes (bottom).

**Figure S5.20**: ^1^H NMR (400 MHz, CDCl_3_) spectrum of diethyl 4-bromopyridine-2,6-dicarboxylate.

Ethyl 4-bromo-6-(hydroxymethyl)picolinate^6^

Diethyl diethyl 4-bromopyridine-2,6-dicarboxylate, (1.23 g, 4.07 mmol, 1 eq.) was dissolved in a solution of DCM (7 ml) and ethanol (9 ml) and cooled in an ice bath. After that, solid sodium borohydride (0.23 g, 6.11 mmol, 1.5 eq.) was slowly added and the resulting solution was stirred at 0 °C for 4 hours under argon, while monitoring the reaction using TLC (silica, 3% MeOH in DCM). Once the starting material was consumed, a solution of 1 M HCl (6 ml) was added to the reaction mixture, followed by water (25 ml) and DCM (20 ml). The resulting aqueous layer was separated and washed with DCM (10 ml × 3). After that, the combined organic layers were washed with water (10 ml × 3), dried over MgSO_4_, filtered and concentrated under reduced pressure. The crude solution was then purified by flash chromatography (silica, DCM to 2.5% MeOH in DCM) to yield white solid (0.675 g, 2.60 mmol, 64%).

LCMS (ESI^+^) *m/z* 260.102 [M+H]^+^.

^1^H NMR (400 MHz, CDCl_3_) *δ* 8.17 (1 H, d, ^4^J_H-H_ 1.83, H^4^), 7.73 (1 H, d, ^4^J_H-H_ 1.83, H^2^), 4.84 (2 H, s, H^6^), 4.46 (2 H, q, ^3^J_H-H_ 6.9, H^8^), 1.43 (6 H, t, ^3^J_H-H_ 7.1, H^9^).

**Figure S5.21**: LCMS (Method E) of ethyl 4-bromo-6-(hydroxymethyl)picolinate AC (210-400 nm) (top) and the MS (ESI^+^) at 2.345 minutes (bottom).

**Figure S5.22**: ^1^H NMR (400 MHz, CDCl_3_) spectrum of ethyl 4-bromo-6-(hydroxymethyl)picolinate.

Ethyl 6-(hydroxymethyl)-4-((2,4,6-trimethoxyphenyl)ethynyl)picolinate

2-ethynyl-1,3,5-trimethoxybenzene, (300 mg, 1.56 mmol, 1 eq.), ethyl 4-bromo-6-(hydroxymethyl)picolinate (446 mg, 1.72 mmol, 1.1 eq.) and [Pd(allyl)Cl]_2_ (60 mg, 0.164 mmol, 0.1 eq.) were dissolved in anhydrous acetonitrile (5 ml) and degassed freeze-pump-thaw × 3). After that, TEA (2.16 ml, 15.6 mmol, 10 eq.) and piperidine (0.6 ml, 6.24 mmol, 4 eq.) were added, and the resulting solution was stirred at 40°C for 12 hours under argon. Organic solvents were then removed under reduced pressure, and the residue was dissolved in DCM (10 ml). The resulting solution was washed with water (5 ml × 3), and the combined aqueous phases were extracted with DCM (5 ml × 3). The combined organic layers were dried over MgSO_4_, filtered and isolated from the solvent under reduced pressure. The crude product was then purified by column chromatography (silica, 4% MeOH in DCM) to yield yellow solid (204 mg, 0.550 mmol, 35%). HRMS (ESI^+^) *m/z* 372.1443 (C_20_H_22_NO_6_ requires 372.1447).

^1^H NMR (600 MHz, CDCl_3_) *δ* 8.10 (1 H, d, ^4^J_H-H_ 1.4, H^4^), 7.58 (1 H, d, ^4^J_H-H_ 1.4, H^2^), 6.11 (2 H, s, H^14^), 4.82 (2 H, s, H^6^), 4.47-4.43 (2H, m, H^8^), 3.90 (6 H, s, H^16^), 3.85 (3 H, s, H^17^), 1.42 ( 3 H, t, ^3^J_H-H_ 7.1, H^9^).

^13^C NMR (151 MHz, CDCl_3_) *δ* 164.86 (C^7^), 162.87 (C^15^), 162.82 (C^13^), 159.92 (C^1^), 147.14 (C^5^), 134.48 (C^3^), 125.61(C^4^), 125.14 (C^2^), 93.35 (C^10^), 93.07 (C^11^), 90.44 (C^14^), 89.26 (C^12^), 64.38 (C^6^), 61.94 (C^8^), 56.07 (C^16^), 55.48 (C^17^), 14.27 (C^9^).

**Figure S5.23**: Accurate MS (ESI^+^) of ethyl 6-(hydroxymethyl)-4-((2,4,6-trimethoxyphenyl)ethynyl)picolinate.

**Figure S5.24**: LCMS (Method E) of ethyl 6-(hydroxymethyl)-4-((2,4,6-trimethoxyphenyl)ethynyl)picolinate AC (210-400 nm) (top) and the MS (ESI^+^) at 2.282 minutes (bottom).

**Figure S5.25**: ^1^H NMR (600 MHz, CDCl_3_) (top) and ^13^C NMR (151 MHz, CDCl_3_) (bottom) spectra of ethyl 6-(hydroxymethyl)-4-((2,4,6-trimethoxyphenyl)ethynyl)picolinate.

Ethyl 6-(((methylsulfonyl)oxy)methyl)-4-((2,4,6-trimethoxyphenyl)ethynyl)picolinate

Ethyl 6-(hydroxymethyl)-4-((2,4,6-trimethoxyphenyl)ethynyl)picolinate (119 mg, 0.320 mmol, 1 eq.) was dissolved in anhydrous THF (3.5 ml). DIPEA (0.17 ml, 0.960, 3 eq.) and methanesulfonic anhydride (0.223 g, 1.280 mmol, 4 eq.) were added, and the resulting mixture was stirred at rt for 1 hour under argon. Once the reaction completion was confirmed by TLC (silica, 5% MeOH in DCM), organic solvents were evaporated under reduced pressure, and the residue was redissolved in DCM (10 ml). The resulting solution was washed with water (10 ml × 3), and the combined aqueous phases were extracted with DCM (5 ml × 3). The combined organic layers were dried over MgSO_4_, filtered and isolated from solvent under reduced pressure to yield pale yellow solid that was used in the next step without further purification.

^1^H NMR (400 MHz, CDCl_3_) *δ* 8.17 (1 H, s, H^4^), 7.72 (1 H, s, H^2^), 6.12 (2 H, s, H^14^), 5.44 (2 H, s, H^6^), 4.58-4.47 (2H, m, H^8^), 3.91 (6 H, s, H^16^), 3.86 (3 H, s, H^17^), 1.44 ( 3 H, t, ^3^J_H-H_ 7.5, H^9^).

**Figure S5.26**: LCMS (Method E) of ethyl 6-(((methylsulfonyl)oxy)methyl)-4-((2,4,6-trimethoxyphenyl) ethynyl)picolinate AC (210-400 nm) (top) and the MS (ESI^+^) at 3.093 minutes (bottom).

**Figure S5.27**: ^1^H NMR (400 MHz, CDCl_3_) spectrum of ethyl 6-(((methylsulfonyl)oxy)methyl)-4-((2,4,6-trimethoxyphenyl)ethynyl)picolinate.

Triethyl 6,6',6''-((1,4,7-triazonane-1,4,7-triyl)tris(methylene))tris(4-((2,4,6-trimethoxyphenyl)ethynyl)picolinate)

Ethyl 6-(((methylsulfonyl)oxy) methyl)-4-((2,4,6-trimethoxyphenyl) ethynyl) picolinate, **15** (*0.144* *g*, *0.320 mmol,* 3.5 eq.), TACN.3HCl (22 mg, 0.0914 mmol, 1 eq.) and K_2_CO_3_ (88 mg, 0.6398 mmol, 7 eq.) were dissolved in anhydrous acetonitrile (5 ml) and stirred at 60°C for 20 hours under argon. The crude was then isolated from solvent under reduced pressure and redissolved in MeOH for the use in the next step without purification.

HRMS (ESI^+^) *m/z* 1189.5195 [M+H]^+^ (C_66_H_72_N_6_O_15_ requires 1189.5134).

**Figure S5.28**: Accurate MS (ESI^+^) of triethyl 6,6',6''-((1,4,7-triazonane-1,4,7-triyl)tris(methylene))tris(4-((2,4,6-trimethoxyphenyl)ethynyl)picolinate).

**EuL^1^**

Triethyl 6,6',6''- ((1,4,7-triazonane-1,4,7-triyl) tris (methylene)) tris (4-((2,4,6-trimethoxyphenyl) ethynyl)picolinate) (*0.0914* mmol) was dissolved in MeOH (5 ml). Water was then drop-wise added to the resulting solution until the white precipitate remained undissolved. The pH of the resulting solution was adjusted to 12.5 using 1M NaOH. The resulting solution was stirred at rt for 72 hours, while its pH was monitored and periodically adjusted to 12.5 until hydrolysis completion was confirmed by LCMS. After that, the solution pH was readjusted to 6.5 using 1 M HCl. EuCl_3_.6H_2_O was then added (37 mg, 0.101 mmol, 1.1 eq.), and the resulting solution was stirred at rt for 18 hours. Solvents were then removed under reduced pressure, and the resulting pale yield yellow solid was purified by reverse-phase HPLC (C18, Method B, retention time = 11.4 minutes) to yield yellow solid (67 mg, 58% over 3 steps).

HRMS (ESI^+^) *m/z* 1253.3190 [M+H]^+^ (C_60_H_57_^151^EuN_6_O_15_ requires 1253.3159).

**Figure S5.29**: Simulated (top) and recorded (bottom) accurate MS (ESI^+^) of **EuL^1^**.

**Figure S5.30**: LCMS (Method E) of **EuL^1^** AC (210-400 nm) (top) and the MS (ESI^+^) at 2.445 minutes (bottom).

2,4,6-Trimethoxy-3-nitrobenzaldehyde

To an ice-cold solution of 2,4,6-trimethoxybenzaldehyde (1.00 g, 5.10 mmol, 1 eq.) in concentrated sulfuric acid (5 ml) the solution of ammonium nitrate (0.450 g. 5.61 mmol, 1.1 eq.) in concentrated sulfuric acid (5 ml) was dropwise added over 10 minutes, and the resulting mixture was poured over ice. The resulting suspension was then filtered, and the solid residue was washed with water (10 ml × 3) and dried to yield beige solid (1.157 g, 4.80 mmol, 94%) which was used in the next step without further purification.

HRMS (ESI^+^) *m/z* 242.0673 (C_10_H_12_NO_6_ requires 242.0673)

^1^H NMR (700 MHz, CDCl_3_) *δ* 10.25 (1H, s, H^1^), 6.31 (1H, s, H^4^), 3.96 (3H, s, H^9^) 3.95 (3H, s, H^8^), 3.91 (3H, s, H^10^).

^13^C NMR (151 MHz, CDCl_3_) *δ* 186.37 (C^1^), 164.86 (C^5^), 156.88 (C^3^), 155.38 (C^7^), 131.22 (C^6^), 111.96 (C^2^), 91.39 (C^4^), 64.74 (C^10^), 56.85 (C^8^), 56.66 (C^9^).

**Figure S5.31**: Accurate MS (ESI^+^) of 2,4,6-trimethoxy-3-nitrobenzaldehyde.

**Figure S5.32**: LCMS (Method E) of 2,4,6-trimethoxy-3-nitrobenzaldehyde AC (210-400 nm) (top) and the MS (ESI^+^) at 1.992 minutes (bottom).

**Figure S5.33**: ^1^H NMR (700 MHz, CDCl_3_) (top) and ^13^C NMR (151 MHz, CDCl_3_) (bottom) spectra of 2,4,6-trimethoxy-3-nitrobenzaldehyde.

2-(2,2-Dibromovinyl)-1,3,5-trimethoxy-4-nitrobenzene

A solution of CBr_4_ (2.07 g, 6.23 mmol, 1.5 eq.) in anhydrous DCM (10 ml) was dropwise added to an ice-cold solution of 2,4,6-trimethoxy-3-nitrobenzaldehyde (1.00 g, 4.15 mmol, 1 eq.) and PPh_3_ (3.27 g, 12.5 mmol, 3 eq.) in anhydrous DCM (5 ml), and the resulting solution was stirred at rt for 20 hours under argon. The crude mixture was then purified by column chromatography (silica, 50% DCM in hexane) to yield beige solid (1.20 g, 3.04 mmol, 73%).

^1^H NMR (700 MHz, CDCl_3_) *δ* 7.19 (1H, s, H^1^), 6.27 (1H, s, H^4^), 3.91 (3H, s H^8^), 3.88 (3H, s, H^9^), 3.82 (3H, s, H^10^).

^13^C NMR (151 MHz, CDCl_3_) *δ* 158.95 (C^5^), 153.10 (C^7^), 151.48 (C^7^), 130.68 (C^6^), 129.14 (C^1^), 111.88 (C^2^), 95.80 (C^11^), 91.34 (C^4^), 62.77 (C^10^), 56.65 (C^8^).

HRMS (ESI^+^) *m/z* 395.9080 (C_11_H_12_Br_2_NO_5_ requires 395.9082)

**Figure S5.34**: Accurate MS (ESI^+^) of 2-(2,2-dibromovinyl)-1,3,5-trimethoxy-4-nitrobenzene.

**Figure S5.35**: LCMS (Method E) of 2-(2,2-dibromovinyl)-1,3,5-trimethoxy-4-nitrobenzene AC (210-400 nm) (top) and the MS (ESI^+^) at 2.888 minutes (bottom).

**Figure S5.36**: ^1^H NMR (700 MHz, CDCl_3_) (top) and ^13^C NMR (151 MHz, CDCl_3_) (bottom) spectra of 2-(2,2-dibromovinyl)-1,3,5-trimethoxy-4-nitrobenzene.

1-Ethynyl-2,4,6-trimethoxy-3-nitrobenzene

2-(2,2-dibromovinyl)-1,3,5-trimethoxy-4-nitrobenzene (1.13 g, 2.83 mmol, 1 eq.) was dissolved in anhydrous THF (10 ml) and cooled to -78 °C (acetone, dry ice). 2.5 M solution of n-butyllithium in hexane (2.30 ml, 5.67 mmol, 2 eq.) was then added dropwise, and the resulting orange solution was stirred for 30 minutes at -78 °C. Water (20 ml) was then added dropwise, and the resulting solution was stirred at rt for another 30 minutes. After that, the organic solvents were removed under reduced pressure, and the resulting aqueous solution was extracted with DCM (20 ml × 3). The combined organic layers were washed with brine (10 ml) and water (15 ml × 3), dried over MgSO_4_ and filtered. DCM solvent was removed under reduced pressure to yield yellow oil that was purified by column chromatography (silica, 30% hexane in DCM) to yield yellow solid (0.163 g, 24%).

HRMS (ESI^+^) *m/z* 238.0718 (C_11_H_12_NO_5_ requires 238.0715)

^1^H NMR (700 MHz, CDCl_3_) *δ* 6.25 (1H, s, H^4^), 4.05 (3H, s, H^10^), 3.94 (3H, s, H^9^), 3.91 (3H, s, H^8^), 3.52 (1H, s, H^11^).

^13^C NMR (151 MHz, CDCl_3_) *δ* 163.53 (C^5^), 156.16 (C^7^), 153.00 (C^3^), 130.61 (C^6^), 98.54 (C^1^), 90.88 (C^4^), 86.34 (C^11^), 74.51 (C^2^), 62.65 (C^10^), 56.60 (C^8^), 56.54 (C^9^).

**Figure S5.37**: Accurate MS (ESI^+^) of 1-ethynyl-2,4,6-trimethoxy-3-nitrobenzene.

**Figure S5.38**: LCMS (Method E) of 1-ethynyl-2,4,6-trimethoxy-3-nitrobenzene AC (210-400 nm) (top) and the MS (ESI^+^) at 2.275 minutes (bottom).

**Figure S5.39**: ^1^H NMR (700 MHz, CDCl_3_) (top) and ^13^C NMR (151 MHz, CDCl_3_) (bottom) spectra of 1-ethynyl-2,4,6-trimethoxy-3-nitrobenzene.

Ethyl 6-(hydroxymethyl)-4-((2,4,6-trimethoxy-3-nitrophenyl)ethynyl)picolinate

2-(2,2-dibromovinyl)-1,3,5-trimethoxy-4-nitrobenzene (150 mg, 0.632 mmol, 1 eq.), ethyl 4-bromo-6-(hydroxymethyl)picolinate (181 mg, 0.695, 1.1 eq.) and [Pd(allyl)Cl]_2_ (23 mg, 0.0632 mmol, 0.1 eq.) were dissolved in anhydrous acetonitrile (3 ml) and degassed (freeze-pump-thaw × 3). After that, TEA (1.00 ml, 7.22 mmol, 11 eq.) and piperidine (0.25 ml, 2.6 mmol, 4 eq.) were added, and the resulting solution was stirred at 40°C for 20 hours under argon). Organic solvents were then removed under reduced pressure, and the resulting yellow solid was purified by column chromatography (silica, 3 % MeOH in DCM) to yield beige solid (0.207 g, 0.497 mmol, 79%).

HRMS (ESI^+^) *m/z* 417.1288 (C_20_H_21_N_2_O_8_ requires 417.1298).

^1^H NMR (700 MHz, CD_2_Cl_2_) *δ* 8.06 (1H, s, H^13^), 7.58 (1H, s, H^16^), 6.33 (1H, s, H^4^), 4.81 (2, q, ^3^J_H-H_ 7.1, H^17^), 4.43 (2H, q, ^3^J_H-H_ 7.1, H^19^), 4.11 (3H, s, H^10^) 3.99 (3H, s, H^9^), 3.95 (3H, s, H^8^), 1.42 (3H, t, ^3^J_H-H_ 7.1, H^20^).

^13^C NMR (151 MHz, CD_2_Cl_2_) *δ* 164.90 (C^18^), 163.85 (C^5^), 160.92 (C^15^), 156.00 (C^7^), 154.16 (C^3^), 148.11 (C^14^), 133.52 (C^12^), 125.49 (C^13^), 125.19 (C^16^), 98.52 (C^1^), 95.15 (C^11^), 91.64 (C^4^), 86.54 (C^2^), 64.67 (C^17^), 63.29 (C^10^), 62.35 (C^19^), 57.23 (C^8^), 57.11 (C^9^), 14.47 (C^20^).

**Figure S5.40**: Accurate MS (ESI^+^) of ethyl 6-(hydroxymethyl)-4-((2,4,6-trimethoxy-3-nitrophenyl) ethynyl)picolinate.

**Figure S5.41**: LCMS (Method E) of ethyl 6-(hydroxymethyl)-4-((2,4,6-trimethoxy-3-nitrophenyl) ethynyl)picolinate AC (210-400 nm) (top) and the MS (ESI^+^) at 2.382 minutes (bottom).

**Figure S5.42**: ^1^H NMR (700 MHz, CDCl_3_) (top) and ^13^C NMR (151 MHz, CDCl_3_) (bottom) spectra of ethyl 6-(hydroxymethyl)-4-((2,4,6-trimethoxy-3-nitrophenyl) ethynyl)picolinate.

Ethyl 6-(((methylsulfonyl)oxy)methyl)-4-((2,4,6-trimethoxy-3-nitrophenyl)ethynyl)picolinate

Ethyl 6-(hydroxymethyl)-4-((2,4,6-trimethoxy-3-nitrophenyl)ethynyl)picolinate (200 mg, 0.480 mmol, 1 eq.) and methanesulfonic anhydride (335 mg, 1.921 mmol, 4 eq.) were dissolved in anhydrous THF (10 ml) and DIPEA (0.250 ml, 1.44 mmol, 3 eq.) was added. The resulting solution was stirred at rt for 1 hour under argon). Once the reaction completion was confirmed by TLC (silica, 5% MeOH in DCM), the volatile organic materials were evaporated under reduced pressure, and the obtained yellow oil was dissolved in DCM (10 ml). The resulting solution was washed with water (10 ml × 3), and the combined aqueous phases were extracted with DCM (5 ml × 3). The combined organic layers were dried over MgSO_4_, filtered and isolated from the solvent under reduced pressure to yield pale yellow solid that was used in the next step without further purification.

LCMS (ESI^+^) *m/z* 495.388 [M+H]^+^

**Figure S5.43**: LCMS (Method E) of ethyl 6-(((methylsulfonyl)oxy)methyl)-4-((2,4,6-trimethoxy-3-nitrophenyl)ethynyl)picolinate AC (210-400 nm) (top) and the MS (ESI^+^) at 2.653 minutes (bottom).

Triethyl 6,6',6''-((1,4,7-triazonane-1,4,7-triyl)tris(methylene))tris(4-((2,4,6-trimethoxy-3-nitrophenyl)ethynyl)picolinate)

Ethyl 6-(((methylsulfonyl)oxy)methyl)-4-((2,4,6-trimethoxy-3-nitrophenyl)ethynyl)picolinate (*0.480 mmol,* 3.5 eq.), TACN.3HCl (32 mg, 0.137 mmol, 1 eq.) and K_2_CO_3_ (133 mg, 0.960 mmol, 7 eq.) were dissolved in anhydrous acetonitrile (5 ml) and stirred at 60°C for 12 hours under argon. The crude was isolated from the solvent under reduced pressure and dissolved in DCM. It was then washed with water (5 ml × 3), and the combined aqueous phases were extracted with DCM (5 ml × 3). The combined organic layers were dried over MgSO_4_, filtered and isolated from the solvent under reduced pressure to yield pale yellow solid (*0.137 mmol*) that was used in the next step without further purification.

HRMS (ESI^+^) *m/z* 1324.4679 [M+H]^+^ (C_66_H_69_N_9_O_21_ requires 1324.4686).

**Figure S5.44**: Accurate MS (ESI^+^) of triethyl 6,6',6''-((1,4,7-triazonane-1,4,7-triyl)tris(methylene))tris(4-((2,4,6-trimethoxy-3-nitrophenyl)ethynyl)picolinate).

**EuL^2^**

Triethyl 6,6',6''-((1,4,7-triazonane-1,4,7-triyl) tris (methylene)) tris (4-((2,4,6-trimethoxy-3-nitrophenyl) ethynyl) picolinate) (*0.137 mmol*) was dissolved in MeOH (5 ml) and water was dropwise added until white precipitate remained undissolved. The pH of the resulting solution was adjusted to 12.5 using 1M NaOH, and it was then stirred at rt for 3 hours, while its pH was periodically adjusted to 12.5 until hydrolysis completion was confirmed by LCMS. After that, the solution pH was readjusted to 6.5 using 1M HCl. Eu(OAC)_3_ was then added (50 mg, 0.151 mmol, 1.1 eq.) and the resulting solution was stirred at rt for 20 hours. The solvent was then removed under reduced pressure to yield yellow solid which was purified by flash chromatography (silica, 5% MeOH in DCM) to yield yellow solid (31 mg, 0.022 mmol, 16% over two steps).

HRMS (ESI^+^) *m/z* 1388.2751 [M+H]^+^ (C_60_H_54_^151^EuN_9_O_21_ requires 1388.2711).

**Figure S5.45**: LCMS (Method E) of **EuL^2^** AC (210-400 nm) (top) and the MS (ESI^+^) at 2.601 minutes (bottom).

**Figure S5.46**: Simulated (top) and recorded (bottom) accurate MS (ESI^+^) of **EuL^2^**.

4-Hydroxy-2,6-dimethoxybenzaldehyde^7^

An ice-cold solution of phosphorus oxychloride (9.0 ml, 97 mmol, 1.5 eq.) in DCM (30 ml) was dropwise added to an ice-cold solution 3,5-dimethoxyphenol (10 g, 65 mmol, 1 eq.) in DCM (30 ml), and the resulting solution was stirred for 10 minutes in an ice bath. To the resulting solution DMF (5.5 ml, 71 mmol, 1.1 eq.) was added dropwise over 40 minutes and stirred for further 1 hour in an ice bath once addition was completed. After that, water (250 ml) was slowly added to the mixture which was then stirred vigorously for 15 minutes at rt. The resulting solution was washed with DCM (100 ml × 3), and the aqueous layer was then basified with 1M NaOH until pH = 6 and then stirred for 10 minutes. The resulting suspension was filtered, and the residue was dried under reduced pressure. The crude product was then recrystallised from ethanol to yield pale-yellow solid (3.9 g, 21 mmol, 32%).

LCMS (ESI+) *m/z* 543.500 [M+H]^+^.

^1^H NMR (400 MHz, MeOD) *δ* 10.14 (1H, s, H^5^), 6.09 (2H, s, H^3^), 3.83 (6H, s, H^6^).

^13^C NMR (100 MHz, MeOD) *δ* 191.61, 187.68, 166.46, 164.80, 91.31, 54.72.

**Figure S5.47**: ^1^H NMR (400 MHz, MeOD) (top) and ^13^C NMR (100 MHz, MeOD) (bottom) spectra of 4-hydroxy-2,6-dimethoxybenzaldehyde.

**Figure S5.48**: LCMS (Method E) of 4-hydroxy-2,6-dimethoxybenzaldehyde AC (210-400 nm) (top) and the MS (ESI^+^) at 1.164 minutes (bottom).

4-(2,2-Dibromovinyl)-3,5-dimethoxyphenol

A solution of CBr_4_ (2.46 g, 7.41 mmol, 1.5 eq.) in anhydrous DCM (30 ml) was dropwise added to an ice-cold solution of 4-hydroxy-2,6-dimethoxybenzaldehyde (900 mg, 4.94, 1 eq.) and PPh_3_ (3.90 g, 14.82 mmol, 3 eq.) in anhydrous DCM (10 ml), and the resulting solution was stirred at rt for 20 hours under argon. After that, the solution was concentrated and the crude was purified by column chromatography (silica, from 2:3 to 1:3 hexane to DCM) to yield pale yellow solid (250 mg, 0.740 mmol, 15%).

HRMS (ESI^+^) *m/z* 336.9081 [M+H]^+^ (C_14_H_18_N_3_O_3_Br_2_ requires 336.9075).

^1^H NMR (400 MHz, CDCl_3_) *δ* 7.16 (1H, s, H^5^), 6.05 (2H, s, H^3^), 3.78 (6H, S, H^7^).

^13^C NMR (100 MHz, CDCl_3_) 158.18, 157.64, 130.82, 106.81, 92.87, 91.90, 55.70.

**Figure S5.49**: Accurate MS (ESI^+^) of 4-(2,2-dibromovinyl)-3,5-dimethoxyphenol.

**Figure S5.50**: LCMS (Method E) of 4-(2,2-dibromovinyl)-3,5-dimethoxyphenol AC (210-400 nm) (top) and the MS (ESI^+^) at 2.608 minutes (bottom).

**Figure S5.51**: ^1^H NMR (400 MHz, CDCl_3_) (top) and ^13^C NMR (100 MHz, CDCl_3_) (bottom) spectra of 4-(2,2-dibromovinyl)-3,5-dimethoxyphenol.

4-Ethynyl-3,5-dimethoxyphenol

4-(2,2-dibromovinyl)-3,5-dimethoxyphenol (240 mg, 0.710 mmol, 1 eq.) was dissolved in anhydrous THF (10 ml) and cooled to -78 °C (acetone, dry ice). 2.5 M solution of n-butyllithium in hexane (1.15 ml, 2.84 mmol, 4 eq.) was then added dropwise, and the resulting orange solution was stirred at -78°C for 20 minutes under argon. Water (20 ml) was then added dropwise, and the resulting solution was stirred at rt for further 20 min. After that, the organic solvents were removed under the reduced pressure, and the crude was recrystallised from MeOH to give orange solid which was used in the next step without further purification.

HRMS (ESI^+^) 179.0721 [M+H]^+^ (C_10_H_11_O_3_ requires 179.0708)

**Figure S5.68**: Accurate MS (ESI^+^) of **29**.

**Figure S5.52**: LCMS (Method E) of 4-ethynyl-3,5-dimethoxyphenol AC (210-400 nm) (top) and the MS (ESI^+^) at 1.583 minutes (bottom).

5-(4-Bromobutoxy)-2-ethynyl-1,3-dimethoxybenzene

A solution of 4-ethynyl-3,5-dimethoxyphenol (*0.710 mmol, 1 eq.*) in DMF (5 ml) was dropwise added over 1 hour to a solution of 1,4-dibromobutane (3 ml, 25.0 mmol, 35 eq.) and K_2_CO_3_ (143 mg, 1.04 mmol, 1.5 eq.) in DMF (5 ml) stirring at 70°C. Once the addition was completed, the resulting solution was stirred for additional 10 minutes before DMF solvent was removed under reduced pressure. The crude product was purified by flash chromatography (silica, 3:7 EtOAc - hexane) to yield pale yellow solid (142 mg, 0.453 mmol, 64% over two steps).

HRMS (ESI^+^) *m/z* 313.04340 [M+H]+ (C_14_H_17_BrO_3_ requires 313.04338).

^1^H NMR (400 MHz, CDCl_3_) *δ* 6.09 (2H, s, H^3^), 4.02 (2H, t, ^3^J_H-H_ 6.1, H^8^), 3.87 (6H, s, H^7^), 3.49 (3H, t, ^3^J_H-H_ 6.5, H^11^), 3.49(1H, s, H^6^) 2.07 (2H, m, H^9^), 1.96 (2H, m, H^10^).

^13^C NMR (100 MHz, CDCl_3_) *δ* 163.02, 161.22, 93.14, 90.84, 83.81, 67.06, 56.11, 33.31, 29.37, 27.79.

**Figure S5.53**: Accurate MS (ESI^+^) of 5-(4-bromobutoxy)-2-ethynyl-1,3-dimethoxybenzene.

**Figure S5.54**: LCMS (Method E) of 5-(4-bromobutoxy)-2-ethynyl-1,3-dimethoxybenzene AC (210-400 nm) (top) and the MS (ESI^+^) at 2.858 minutes (bottom).

**Figure S5.55**: ^1^H NMR (400 MHz, CDCl_3_) (top) and ^13^C NMR (100 MHz, CDCl_3_) (bottom) spectra of 5-(4-bromobutoxy)-2-ethynyl-1,3-dimethoxybenzene.

5-(4-Azidobutoxy)-2-ethynyl-1,3-dimethoxybenzene

5-(4-bromobutoxy)-2-ethynyl-1,3-dimethoxybenzene (122 mg, 0.390 mmol, 1 eq.), sodium azide (100 mg, 1.54 mmol, 4 eq.) and sodium iodide (58 mg, 0.39, 1 eq.) were dissolved in DMF (10 ml) and stirred for 20 hours at rt. The resulting mixture was then isolated from DMF solvent under reduced pressure and redissolved in DCM (50 ml). The resulting solution was washed with water (20 ml × 3), and the combined aqueous fractions were extracted with DCM. The combined organic fractions were dried over MgSO_4_ and filtered. The product was isolated from DCM solvent under reduced pressure to give pale-yellow solid (98 mg, 0.356 mmol, 91%).

HRMS (ESI^+^) *m/z* 276.1345 [M+H]^+^ (C_14_H_18_N_3_O_3_ requires 276.1348).

**Figure S5.56**: Accurate MS (ESI^+^) of 5-(4-azidobutoxy)-2-ethynyl-1,3-dimethoxybenzene.

**Figure S5.57**: LCMS (Method E) of 5-(4-azidobutoxy)-2-ethynyl-1,3-dimethoxybenzene AC (210-400 nm) (top) and the MS (ESI^+^) at 2.667 minutes (bottom).

Ethyl 4-((4-(4-azidobutoxy)-2,6-dimethoxyphenyl)ethynyl)-6-(hydroxymethyl)picolinate

5-(4-azidobutoxy)-2-ethynyl-1,3-dimethoxybenzene (75 mg, 0.272 mmol, 1 eq.) ethyl 4-bromo-6-(hydroxymethyl)picolinate (73 mg, 0.281 mmol, 1.03 eq.) and [Pd(allyl)Cl]_2_ (10 mg, 0.027 mmol, 0.1 eq.) were dissolved in anhydrous acetonitrile (2 ml) and degassed (freeze-pump-thaw × 3). After that, TEA (0.380 ml, 2.70 mmol, 10 eq.) and piperidine (0.105 ml, 1.09 mmol, 4 eq.) were added, and the resulting solution was stirred at 40°C for 20 h under argon. Organic solvents were then removed under reduced pressure, and the resulting yellow solid was purified by column chromatography (silica, 3% MeOH in DCM) to yield yellow solid (56 mg, 0.123 mmol, 45%).

HRMS (ESI^+^) *m/z* 455.1914 [M+H]^+^ (C_14_H_17_BrO_3_ requires 455.1931).

^1^H NMR (700 MHz, CDCl_3_) *δ* 8.11 (1H, s, H^4^), 7.57 (1H, s, H^2^), 6.11 (2H, s, H^14^), 4.83 (2H, s, H^6^), 4.47 (2H, q, ^3^J_H-H_ 7.2 H^8^), 4.04 (2H, t, ^3^J_H-H_ 6.0, H^17^), 3.90 (6H, s, H^16^), 3.39 (2H, t,  ^3^J_H-H_ 7.0, H^20^), 1.90 (2H, m, H^18^), 1.82 (2H, m, H^19^), 1.44 (3H, t, ^3^J_H-H_ 7.3, H^9^)

^13^C NMR (151 MHz, CDCl_3_) *δ* 164.94 (C^7^), 162.99 (C^13^), 162.33 (C^15^), 159.91 (C^1^), 147.24 (C^5^), 134.74 (C^3^), 125.84 (C^4^), 125.30 (C^2^), 93.35 (C^10^), 93.27 (C^12^), 91.03 (C^14^), 89.59 (C^11^), 67.61 (C^17^), 64.46 (C^6^), 62.15 (C^8^), 56.26 (C^16^), 51.25 (C^20^), 26.55 (C^18^), 25.84 (C^19^), 14.45 (C^9^).

**Figure S5.58**: Accurate MS (ESI^+^) of ethyl 4-((4-(4-azidobutoxy)-2,6-dimethoxyphenyl)ethynyl)-6-(hydroxymethyl)
picolinate.

**Figure S5.59**: LCMS (Method E) of ethyl 4-((4-(4-azidobutoxy)-2,6-dimethoxyphenyl)ethynyl)-6-(hydroxymethyl)
picolinate AC (210-400 nm) (top) and the MS (ESI^+^) at 2.800 minutes (bottom).

**Figure S5.60**: ^1^H NMR (700 MHz, CDCl_3_) (top) and ^13^C NMR (151 MHz, CDCl_3_) (bottom) spectra of ethyl 4-((4-(4-azidobutoxy)-2,6-dimethoxyphenyl)ethynyl)-6-(hydroxymethyl)picolinate.

Ethyl 4-((4-(4-azidobutoxy)-2,6-dimethoxyphenyl)ethynyl)-6-(((methylsulfonyl)oxy)methyl)picolinate

Ethyl 4-((4-(4-azidobutoxy)-2,6-dimethoxyphenyl)ethynyl)-6-(hydroxymethyl)picolinate

(32 mg, 0.071 mmol, 1 eq.) and methanesulfonic anhydride (44 mg, 0.25 mmol, 3.5 eq.) were dissolved in anhydrous THF (7 ml) and DIPEA (37 μl, 0.212 mmol, 3 eq.) was added. The resulting solution was stirred at rt for 1 hour under argon. After that, volatile organic materials were evaporated under reduced pressure, and the obtained yellow oil was redissolved in DCM (10 ml). The resulting solution was washed with water (10 ml × 3), and the combined aqueous layers were extracted with DCM (5 ml × 3). The combined organic layers were dried over MgSO_4_ and filtered. DCM solvent was evaporated under reduced pressure to yield pale yellow solid that was used in the next step without further purification.

HRMS(ESI^+^) *m/z* 533.16950 [M+H]^+^ (C_24_H_28_N_4_O_8_S requires 533.17006)

**Figure S5.61**: Accurate MS (ESI^+^) of ethyl 4-((4-(4-azidobutoxy)-2,6-dimethoxyphenyl)ethynyl)-6-(((methylsulfonyl)oxy)methyl)picolinate.

**Figure S5.62**: LCMS (Method E) of ethyl 4-((4-(4-azidobutoxy)-2,6-dimethoxyphenyl)ethynyl)-6-(((methylsulfonyl)oxy)methyl)picolinate AC (210-400 nm) (top) and the MS (ESI^+^) at 3.108 minutes (bottom).

Triethyl 6,6',6''-((1,4,7-triazonane-1,4,7-triyl)tris(methylene))tris(4-((4-(4-azidobutoxy)-2,6-dimethoxyphenyl)ethynyl)picolinate)

K_2_CO_3_ (68 mg, 0.49 mmol, 7 eq.), ethyl4-((4-(4-azidobutoxy)-2,6-dimethoxyphenyl)ethynyl)-6 (((methylsulfonyl)oxy)methyl)picolinate (*0.071 mmol,* 3.5 eq.) and TACN.3HCl (4.8 mg, 0.020 mmol, 1 eq.) were dissolved in MeCN (5 ml) and stirred at 60°C for 12 h under argon. After that, MeCN was removed under reduced pressure, and the crude was redissolved in MeOH and used in the next step without further purification.

HRMS (ESI^+^) *m/z* 1438.6527 [M+H]^+^ (C_75_H_87_N_15_O_15_ requires 1438.6584).

**Figure S5.63**: Accurate MS (ESI^+^) of triethyl 6,6',6''-((1,4,7-triazonane-1,4,7-triyl)tris(methylene))tris(4-((4-(4-azidobutoxy)-2,6-dimethoxyphenyl)ethynyl)picolinate).

**EuL^3^**

Triethyl 6,6',6''-((1,4,7-triazonane-1,4,7-triyl)tris(methylene)) tris(4-((4-(4-azidobutoxy)-2,6-dimethoxyphenyl)ethynyl)picolinate) (*0.020 mmol*) was dissolved in MeOH (5 ml) and water was dropwise added until the precipitate remained undissolved. The pH of the resulting solution was adjusted to 12.5 using 1M NaOH. The solution was stirred at 60 °C for 3 hours while its pH was periodically adjusted to 12.5 until hydrolysis completion was confirmed by LCMS. After that, the solution pH was readjusted to 6.5 using 1 M HCl, and EuCl_3_.6HCl was added (7.5 mg, 0.020 mmol, 1 eq.). The resulting solution was stirred at rt for 20 hours. After that, the solvent was removed under pressure to yield yellow solid which was purified by reverse-phase HPLC (C_18_, Method B, retention time = 14.3 minutes) to yield yellow solid (6.8 mg, 0.0045 mmol, 23% over two steps).

HRMS (ESI^+^) 1502.4485 [M+H^+^] (C_69_H_72_^151^EuN_15_O_15_ requires 1502.4609)

**Figure S5.64**: Simulated (top) and recorded (bottom) accurate MS (ESI^+^) of **EuL^3^**.

**Figure S5.65**: LCMS (Method E) of **EuL^3^** AC (210-400 nm) (top) and the MS (ESI^+^) at 3.258 minutes (bottom).

1-Propargyl-β-D-glucopyranoside

2-propynyl-tetra-O-acetyl-β-D-glucopyranoside (50 mg, 0.055 mmol) was added to a 0.1 M solution of NaOMe in MeOH (3 ml) and stirred at rt for 1 hr. After that, the reaction mixture was neutralised using *Amberlite* H^+^ resin and filtered. MeOH was then removed under reduced pressure to yield white solid (12 mg, 0.054 mmol, 98%).

^1^H NMR (400 MHz, MeOD) 4.50 (1H, d,^3^J_H-H_ 7.9, H^1^), 4.46 (2H, t, ^3^J_H-H_ 2.8, H^7^), 3.92 (1H, d, ^2^J_H-H_ 12.2, H^6^), 3.71 (1H, dd, ^3^J_H-H_ 12.2, ^3^J_H-H_ 5.7, H^6’^), 3.40 (1H, t, ^3^J_H-H_ 8.4, H^3^), 3.32 (1H, m, H^4^), 3.31 (1H, m, H^5^), 3.23 (1H, t, ^3^J_H-H_ 7.5, H^2^), 2.90(1H, t, ^4^J_H-H_ 2.4, H^8^).

**Figure S5.66**: ^1^H NMR (400 MHz, CDCl_3_) spectrum of 1-propargyl-β-D-glucopyranoside.

**EuL^3g^**

To a solution of 1-propargyl-β-D-glucopyranoside, **34** (12 mg, 0.0054 mmol, 4.2 eq.) and **EuL^3^** (2.0 mg, 0.0013 mmol, 1 eq.) in MeCN (5 ml) was added [Cu(MeCN)_4_]PF_6_ (1.0 mg, 0.0027 mmol, 2 eq.). The resulting solution was stirred at rt for 20 hours under argon. After that, water was added (5 ml), and the reaction mixture was stirred for further 30 minutes. MeCN was then evaporated and the resulting suspension was centrifuged (6000 rpm). Once water was removed, the residue was resuspended in MeOH and centrifuged again. The resulting product solution was then separated from the grey residue, and MeOH was removed under reduced pressure to yield yellow solid (2.6 mg, 0.0012 mmol, 92%).

HRMS (ESI^+^) 1078.8431 [M+2H]^2+^ (C_96_H_114_^151^EuN_15_O_33_ requires 1078.8529)

**Figure S5.67**: Simulated (top) and recorded (bottom) accurate MS (ESI^+^) of **EuL^3g^** ([M+2H]^2+^, *m*/2)

**Figure S5.68**: LCMS (Method E) of **EuL^3g^** AC (210-400 nm) (top) and the MS (ESI^+^) at 1.742 minutes (bottom).

Ethyl 4-(4-ethynyl-3,5-dimethoxyphenoxy)butanoate

A solution of 4-ethynyl-3,5-dimethoxyphenol (*0.864 mmol, 1 eq*.) in DMF (5 ml) was dropwise added over 1 hour to a solution of 4-bromobutyrate (1.24 ml, 8.64 mmol,10 eq.) and K_2_CO_3_ (143 mg, 1.04 mmol, 1.5 eq.) in DMF (5 ml) stirring at 60°C. Once the addition was completed, the resulting solution was stirred for additional 10 minutes before DMF solvent was removed under reduced pressure. The crude product was purified by flash chromatography (silica, 3:7 EtOAc - hexane) to yield pale yellow solid (58 mg, 0.198 mmol, 23% over two steps).

HRMS (ESI^+^) *m/z* 293.14148 [M+H]+ (C_25_H_30_NO_8_ requires 293.13835).

^1^H NMR (700 MHz, CDCl_3_) *δ* 6.08 (2H, s, H^3^), 4.12 (2H, q, ^3^J_H-H_ 7.2, H^12^), 4.02 (2H, t, ^3^J_H-H_ 6.1, H^8^), 3.86 (6H, s, H^7^), 3.47 (1H, s, H^6^), 2.50 (2H, t, ^3^J_H-H_ 7.2, H^10^), 2.10 (2H, m, H^9^), 1.25 (3H, t, ^3^J_H-H_ 7.2, H^13^)

^13^C NMR (151 MHz, CDCl_3_) *δ* 173.18 (C^11^), 163.08 (C^2^), 161.27 (C^4^), 93.13 (C^5^), 90.93 (C^3^), 83.86 (C^6^), 76.65 (C^1^) 67.01 (C^8^), 60.61 (C^12^), 56.17 (C^7^), 30.68 (C^10^), 24.57 (C^9^), 14.33 (C^13^).

**Figure S5.69**: Accurate MS (ESI^+^) of ethyl 4-(4-ethynyl-3,5-dimethoxyphenoxy)butanoate.

**Figure S5.70**: LCMS (Method E) of ethyl 4-(4-ethynyl-3,5-dimethoxyphenoxy)butanoate AC (210-400 nm) (top) and the MS (ESI^+^) at 2.473 minutes (bottom).

**Figure S5.71**: ^1^H NMR (700 MHz, CDCl_3_) (top) and ^13^C NMR (151 MHz, CDCl_3_) (bottom) spectra of ethyl 4-(4-ethynyl-3,5-dimethoxyphenoxy)butanoate.

Ethyl 4-((4-(4-ethoxy-4-oxobutoxy)-2,6-dimethoxyphenyl)ethynyl)-6-(hydroxymethyl)picolinate

Ethyl 4-(4-ethynyl-3,5-dimethoxyphenoxy)butanoate (58 mg, 0.12 mmol, 1 eq.), diethyl 4-bromopyridine-2,6-dicarboxylate (98 mg, 0.14 mmol, 1.1 eq.) and [Pd(allyl)Cl]_2_ (8.0 mg, 0.022 mmol, 0.2 eq.) were dissolved in anhydrous acetonitrile (10 ml) and degassed (freeze-pump-thaw × 3). After that, TEA (1.00 ml, 7.22 mmol, 60 eq.) and piperidine (0.25 ml, 2.6 mmol, 22 eq.) were added, and the resulting solution was stirred at 40°C for 20 hours under argon. After that, all volatile materials were removed under reduced pressure, and the crude was purified by reverse-phase HPLC (Method D, retention time = 10.3 min) to give pale-yellow solid (20 mg, 0.042 mmol, 35%).

HRMS (ESI^+^) *m/z* 472.1962 [M+H]^+^ (C_25_H_30_NO_8_ requires 472.1971).

^1^H NMR (400 MHz, CDCl_3_) *δ* 8.11 (1H, s, H19), 7.57 (1H, s, H15), 6.12 (2H, s, H3), 4.82 (2H, s, H19), 4.47 (2H, q, ^3^J_H-H_ 7.1, H21) 4.17 (2H, q, ^3^J_H-H_ 7.2, H^12^), 4.06 (2H, t, ^3^J_H-H_ 6.3, H^8^), 3.90 (6H, s, H^7^), 2.53 (2H, t, ^3^J_H-H_ 7.2, H^10^), 2.13 (2H, m, H^9^), 1.44 (3H, t, ^3^J_H-H_ 7.2, H^22^) 1.25 (3H, t, ^3^J_H-H_ 7.2, H^13^)

^13^C NMR (151 MHz, CDCl_3_) *δ* 173.18 (C^11^), 163.08 (C^2^), 161.27 (C^4^), 93.13 (C^5^), 90.93 (C^3^), 83.86 (C^6^), 76.65 (C^1^) 67.01 (C^8^), 60.61 (C^12^), 56.17 (C^7^), 30.68 (C^10^), 24.57 (C^9^), 14.33 (C^13^).

**Figure S5.72**: Accurate MS (ESI^+^) of ethyl 4-((4-(4-ethoxy-4-oxobutoxy)-2,6-dimethoxyphenyl)ethynyl)-6-(hydroxymethyl)picolinate.

**Figure S5.73**: LCMS (Method E) of ethyl 4-((4-(4-ethoxy-4-oxobutoxy)-2,6-dimethoxyphenyl)ethynyl)-6-(hydroxymethyl)picolinate AC (210-400 nm).

**Figure S5.74**: ^1^H NMR (700 MHz, CDCl_3_) (top) and ^13^C NMR (151 MHz, CDCl_3_) (bottom) spectra of ethyl 4-((4-(4-ethoxy-4-oxobutoxy)-2,6-dimethoxyphenyl)ethynyl)-6-(hydroxymethyl)picolinate.

Ethyl 4-((4-(4-ethoxy-4-oxobutoxy)-2,6-dimethoxyphenyl)ethynyl)-6-(((methylsulfonyl)oxy)methyl)picolinate

ethyl 4-((4-(4-ethoxy-4-oxobutoxy)-2,6-dimethoxyphenyl)ethynyl)-6(hydroxymethyl)picolinate (20 mg, 0.042 mmol, 1 eq.) and methanesulfonic anhydride (27 mg, 0.155 mmol, 3.7 eq.) were dissolved in anhydrous THF (4 ml) and DIPEA (22 μl, 0.126 mmol, 3 eq.) was added. The resulting solution was stirred at rt for 1 hour under argon. After that, volatile organic materials were evaporated under reduced pressure, and the obtained yellow solid was redissolved in DCM (10 ml). The resulting solution was washed with water (10 ml × 3), and the combined aqueous phases were extracted with DCM (5 ml × 3). The combined organic layers were dried over MgSO_4_, filtered, and DCM was evaporated under reduced pressure to yield pale yellow solid that was used in the next step without further purification.

HRMS(ESI^+^) *m/z* 550.1744 [M+H]^+^ (C_26_H_32_NO_10_S requires 550.1747)

**Figure S5.75**: Accurate MS (ESI^+^) of **37**.

**Figure S5.76:** LCMS (Method E) of ethyl 4-((4-(4-ethoxy-4-oxobutoxy)-2,6-dimethoxyphenyl)ethynyl)-6-(((methylsulfonyl)oxy)methyl)picolinate AC (210-400 nm) (top) and the MS (ESI^+^) at 2.900 minutes (bottom).

**EuL^4^** ligand precursor

Potassium carbonate (38 mg, 0.275 mmol, 7 eq.), ethyl 4-((4-(4-ethoxy-4-oxobutoxy)-2,6-dimethoxyphenyl)ethynyl)-6-(((methylsulfonyl)oxy)methyl)picolinate (*0.042 mmol,* 1.1 eq.) and TACN•3HCl (9.2 mg, 0.039 mmol, 1 eq.) were dissolved in MeCN
(5 ml) and stirred at 60°C for 20 hours under argon. After that ethyl 6-(((methylsulfonyl)oxy)methyl)-4-((2,4,6-trimethoxyphenyl)ethynyl)picolinate (0.085 mmol, 2.2 eq.) was dissolved in MeCN (5 ml) and added to the reaction mixture. After that, the mixture was stirred at 60°C for additional 20 hours under argon. After that, MeCN was removed under reduced pressure, and the crude mixture of products was redissolved in MeOH (5 ml) and used in the next step without further purification.

LCMS (ESI^+^) *m/z* 1289.739 [M+H]^+^

**Figure S5.77**: LCMS (Method E) of **EuL^4^** ligand precursor AC (210-400 nm) (top) and the MS (ESI^+^) at 3.158 minutes (bottom).

**EuL^4^**

To a solution of **EuL^4^ ligand precursor** (*0.039* *mmol*) in MeOH (5 ml) was dropwise added water until white precipitate remained. pH of the resulting solution was adjusted to 12.5 using 1M NaOH. The solution was stirred at 60 °C for 3 hours while its pH was periodically adjusted to 12.5 until hydrolysis completion was confirmed by LCMS. After that, the solution pH was readjusted to 6.5 using 1 M HCl. EuCl_3_.6HCl was then added (15 mg, 0.041 mmol, 1 eq.) and the resulting solution was stirred at rt for 20 hours. After that, the solvent was removed under pressure to yield yellow solid which was purified by reverse-phase HPLC (C_18_, Method C, retention time = 20.7 min) to yield yellow solid (3.1 mg, 0.0023 mmol, 6% over two steps).

HRMS (ESI^+^) 1325.3356 [M+H^+^] (C_63_H_61_^151^EuN_6_O_17_ requires 1325.3370)

**Figure S5.78**: Simulated (top) and recorded (bottom) accurate MS (ESI^+^) of **EuL^4^**.

**Figure S5.79**: LCMS (Method E) of **EuL^4^** AC (210-400 nm) (top) and the MS (ESI^+^) at 2.900 minutes (bottom).

Supplementary References

1	R. Carr, R. Puckrin, B. K. McMahon, R. Pal, D. Parker and L.-O. Pålsson, *Methods Appl. Fluoresc.*, 2014, **2**, 024007.

2	L. Arrico, L. Di Bari and F. Zinna, *Chem. Eur. J.*, 2021, **27**, 2920–2934.

3	D. F. De Rosa, P. Stachelek, D. J. Black and R. Pal, *Nat Commun*, 2023, **14**, 1537.

4	A. T. Frawley, R. Pal and D. Parker, *Chem. Commun.*, 2016, **52**, 13349–13352.

5	H. Xu, Q. Wang, Z. Qi, X. Li, Y. Lei, H. An, H. Tian and D. Qu, *Angew Chem Int Ed*, 2025, **64**, e202420707.

6	R. Lengacher, K. E. Martin, D. Śmiłowicz, H. Esseln, P. Lotlikar, A. Grichine, O. Maury and E. Boros, *J. Am. Chem. Soc.*, 2023, **145**, 24358–24366.

7	U. C. Dyer, D. A. Henderson, M. B. Mitchell and P. D. Tiffin, *Org. Process Res. Dev.*, 2002, **6**, 311–316.
